# Spatial control of doping in conducting polymers enables complementary, conformable, implantable internal ion-gated organic electrochemical transistors

**DOI:** 10.1038/s41467-024-55284-w

**Published:** 2025-01-09

**Authors:** Duncan J. Wisniewski, Liang Ma, Onni J. Rauhala, Claudia Cea, Zifang Zhao, Alexander Ranschaert, Jennifer N. Gelinas, Dion Khodagholy

**Affiliations:** 1 Department of Electrical Engineering, University of California, Irvine, CA USA; 2 Department of Electrical Engineering, Columbia University, New York, NY USA; 3 Department of Biomedical Engineering, Columbia University, New York, NY USA; 4 Department of Neurology, Columbia University Medical Center, New York, NY USA; 5 Department of Anatomy and Neurobiology, University of California, Irvine, CA USA; 6 Department of Pediatrics, University of California, Irvine, CA USA; 7 Children’s Hospital of Orange County, Orange, CA USA

**Keywords:** Materials science, Engineering, Nanoscience and technology, Neuroscience

## Abstract

Complementary transistors are critical for circuits with compatible input/output signal dynamic range and polarity. Organic electronics offer biocompatibility and conformability; however, generation of complementary organic transistors requires introduction of separate materials with inadequate stability and potential for tissue toxicity, limiting their use in biomedical applications. Here, we discovered that introduction of source/drain contact asymmetry enables spatial control of de/doping and creation of single-material complementary organic transistors from a variety of conducting polymers of both carrier types. When integrated with the vertical channel design and internal ion reservoirs of internal ion-gated organic electrochemical transistors, we produced matched complementary IGTs (cIGTs) that formed high-performance conformable amplifiers with 200 V/V uniform gain and 2 MHz bandwidth. These amplifiers showed long-term in vivo stability, and their miniaturized biocompatible design allowed implantation in developing rodents to monitor network maturation. cIGTs expand the use of organic electronics in standard circuit designs and enhance their biomedical potential.

## Abstract (web summary)

Complementary transistors are critical for multistage circuits. Asymmetry of source/drain contacts enables creation of single-material, matched complementary organic amplifiers with long-term in vivo stability

## Introduction

Si-based electronics are broadly used and efficiently integrated, primarily due to their: (i) ability to perform a broad range of functions with a single material, (ii) scalability of fabrication processes, and (iii) widely accessible and standardized circuit design tools and foundry protocols. In particular, the latter allowed development of a large repository of circuits over the course of several decades that serves as functional building blocks for various applications^1^. Medical devices and implantable bioelectronics are examples of such applications that are being increasingly used to enable discovery science as well as advanced diagnostics and therapeutics for human disease^2–4^. However, current Si-based devices are not suitable for creating a direct interface with the body because: i) they are hard and rigid, causing a large mechanical mismatch across the abiotic/biotic interface^4^; ii) they have minimal, surface-based electrical interaction with ions, which are the main charge carriers in biological tissue^5^; and iii) they require rigorous encapsulation to be protected from body fluids due to risk of damage from ion penetration and enzymatic reactions^6^. Therefore, it is critical to develop soft and biocompatible transistors that can safely and efficiently transduce, amplify, and process the ionic signals of biological tissue^7^. Organic electronics, and in particular mixed conducting polymers, are uniquely qualified for such applications because they are mechanically soft and biocompatible^8–10^, and they can establish a high charge capacity electrical interface with biological tissue due to their volumetric capacitance^11^. They can also directly interact with electrolyte to establish a mixed-conducting interface where ionic fluctuations in the tissue can create electronic carrier changes across the entire bulk of the polymer^12^. Importantly, mixed conducting polymers can form the channel of high transconductance organic electrochemical transistors (OECTs) and function as first stage amplifiers, increasing the signal to noise ratio of weak physiological signals^13,14^.

To enable organic electrochemical transistors to form implantable circuitry akin to Si-based devices, several obstacles have to be overcome. In order for OECTs to function in a circuit, they require an independent gate electrode. However, OECTs are primarily operated using an Ag/AgCl electrode immersed in the electrolyte, preventing selective gating of transistors^15^. External electrolyte is an integral part of such a transistor, which further complicates independent control in circuits comprised of multiple transistors^16^. The contribution of external electrolyte to transistor operation also fundamentally limits the operating speed of the device, due to its reliance on ion mobility^17–19^. Internal ion-gated organic electrochemical transistors (IGTs) contain mobile ion reservoirs within their channel that significantly reduce the ionic transit time and allow MHz range operation^14,20–22^. In addition, each transistor has its own dedicated gate electrode, permitting selective, cross-talk free operation of densely packed transistors in an integrated circuit^22^. However, to be optimally accessible for creation of complex, functional circuits, it would be beneficial for such electrochemical transistors to follow the established electronic design principles of Si-based circuits. A key enabling design principle of such circuits is their ability to form functional blocks with compatible input/output signal dynamic range and polarity. This property is usually accomplished by utilizing two types of transistors (n- and p-type) that address signals of both polarities (1^st^ and 3^rd^ quadrant)^23^. This complementary design can also lead to more power-efficient digital circuits because both transistors are in the ‘ON’ state only during logic transition periods. Complementary metal oxide semiconductor (CMOS) transistors are generated by controlling the doping state of the Si-based channel to enable electron or hole conduction. Because Si can be used to create both n- and p-type transistors, fabrication of “matched” devices is significantly simplified and efficient signal cascading is possible. In contrast, most approaches to developing n- and p-type electrochemical transistors have focused on leveraging separate electron and hole conducting materials^15,24–26^. These innovations have enabled OECT-based electrophysiological amplifiers^13,22,27,28^, as well as neuromorphic and artificial neurons^29–33^. However, the substantial differences in materials used for n- and p-type OECTs in regards to mobility, carrier density, stability, patternability, and solvent compatibility pose major challenges to creating matched devices while ensuring appropriate long-term electrical performance and scalability^34–37^. Optimally, similar to Si-based CMOS, complementary organic transistors should be made with the same material and enable effective saturation in both 1^st^ (positive drain current and voltages) and 3^rd^ (negative drain current and voltages) operating quadrants. It has been shown that it is possible to establish a rectifying response by enlarging the area of the higher potential contact in a two-terminal device with a mixed conducting polymer channel^38,39^. This nonlinear response arises due to formation of an arrangement that resembles a diode-attached transistor, where the large area positive potential contact acts as a gate and can effectively dedope the channel. Furthermore, this mechanism has been investigated in PEDOT:PSS-based OECTs to improve the saturation region^40^.

Here, we show that modulation of spatial dedoping across a mixed conducting polymer channel enables directional control of channel current and thereby permits generation of complementary internal ion-gated organic electrochemical transistors (cIGTs) using a single organic material. This modulation is accomplished by introducing asymmetric contact areas, which leads to contact-mediated dedoping in the region of the lowest potential contact as revealed by optical moving-front experiments, gate-less operation, and impedance spectroscopy. The enhanced localized dedoping can be targeted to increase saturation regions in the first and third quadrants without requiring any material alterations. This approach can be leveraged for transistors with both horizontal and vertical channels as long as the contact areas are larger than the channel. We also demonstrate the generalizability of our design principle by replicating the process with several conducting polymers including both carrier types and polarities. Using the design rules derived from geometrical variation of the devices, we created highly matched vertical transistors as building blocks of conformable amplifiers. These amplifiers can operate with uniform >200× gain at frequencies exceeding 2 MHz. They exhibited stable performance when chronically implanted in freely moving rats for over one month. Currently available amplifiers cannot be fully implanted to provide high, local voltage amplification in concert with a conformable, miniaturized physical footprint. Although this limitation can be managed in large animal models by incorporating rigid, extruding components, it is a barrier to clinical translation because implantation impedes normal activities or requires a large-scale surgical procedure^41,42^. These translational difficulties are mirrored when attempts are made to perform electrophysiologic investigations into small, immature animals. These fragile organisms, which require ongoing maternal care, cannot tolerate rigid, tethered implants that impair behavior or an extensive surgical procedure^43^. Thus, we tested the translational potential of cIGTs by implanting them in developing mice. cIGT-based voltage amplifiers were completely implanted in these mice, enabling longitudinal electrophysiologic recording in naturalistic environments that was not previously possible. cIGTs are able to form fully functional, complementary circuits based on a single organic material that expresses biologic tissue compatibility and conformability.

## Results

De/doping of transistor channels occurs when a drift potential causes ions to move in the channel. Typically, this drift potential is applied via the gate, but for transistors with miniaturized channels the contact areas become significantly larger than the channel area. We hypothesized that in this geometric configuration, the potential of the contacts could also drift ions and influence the doping state of the channel. Furthermore, if contact areas were asymmetric, it could be possible to control the location where de/doping occurs and thereby optimize saturation in different quadrants.

**Fig. 1:**
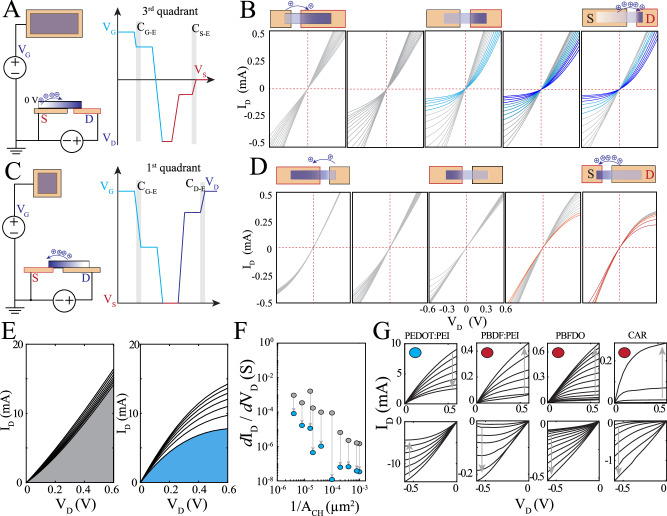
Asymmetrical contact area enables 1^st^ and 3^rd^ quadrant operation independent of electronic carrier type. **A** Schematic of a p-type, asymmetrical, horizontal channel IGT being dedoped via drift potential of gate and source electrodes. **B** Output characteristics of PEDOT:PSS-based IGTs with asymmetric source to drain contact area ratios (A_S_/A_D_ = 1/250, 1/40, 1/1, 40/1, 250/1; from left to right) demonstrating enhanced 3^rd^ quadrant operation, measured with a substantially larger gate electrode area compared to channel area (V_D_, V_G_ = −0.6 to +0.6 V; darker shades represent higher gate voltages; W, L = 100 µm, A_G_ = 250k µm^2^). **C** Schematic of an n-type, asymmetrical, horizontal channel IGT being dedoped via drift potential of gate and source electrodes. **D** Output characteristic of PEDOT:PSS-based IGTs with asymmetric contact areas of source to drain electrodes (A_S_/A_D_ = 250/1 40/1 1/1 1/40 1/250 from left to right) demonstrating 1^st^ quadrant saturation (V_D_, V_G_ = −0.6 to +0.6 V; darker shades represent higher gate voltages; W, L = 100 µm, A_G_ = 100 µm^2^). **E** Comparison of output characteristics of 1^st^ quadrant operating symmetrical (gray) and asymmetrical (blue) contact area transistors with similar channel geometry (A_S_ / A_D_ = 100, W, L = 100, 10 µm). **F** Scatter plot demonstrating scalable enhancement of saturation region slope by utilization of asymmetrical contact area. Gray and blue circles are symmetrical and asymmetrical devices, respectively. **G** Demonstration of 1^st^ and 3^rd^ quadrant operating transistors independent of channel material and electronic carrier type (poly(3,4-ethylenedioxythiophene) polyethylenimine, n-doped poly(benzodifurandione) polyethylenimine, poly(benzodifurandione), and contorted acene nanoribbon. W, L = 100, 10 µm, A_S_/A_D_ = 1/100 (top) or A_S_/A_D_ = 100 (bottom), V_D_, arrow shows the direction of the current traces from V_G_ = −0.6 to +0.6 V). The red and blue circles indicate n- and p-type electronic carriers of the channel material, respectively.

To test this hypothesis, we created a series of depletion-mode transistors using poly(3,4-ethylenedioxythiophene) polystyrene sulfonate (PEDOT:PSS) with source and drain contacts that had up to 3 orders of magnitude difference in area while maintaining the same channel length and width (Fig. 1A, left; Supplementary Fig. 1A, B). We fabricated these transistors in horizontal architecture to accommodate this large geometrical variation. We observed that transistors that had an asymmetric configuration with drain contact area (A_D_) smaller than source contact area (A_S_) exhibited significantly enhanced saturation in the 3^rd^ quadrant (Fig. 1A, B). To systematically quantify the saturation region, we compared the derivative of the drain current to drain voltage across various geometries (Supplementary Fig. 2A, B). We found that reducing the area of the lowest potential contact, in this case the drain (V_D_ < 0), led to enhanced drain current (I_D_) saturation (Supplementary Fig. 2C). These results suggest that the asymmetry between the drain and source contacts increases localized channel dedoping sufficiently to modulate transistor output characteristics. In all cases, the transistors had gate areas that were substantially larger than contact areas. Gate potential (V_G_ ≥ 0 V) remained higher than source (V_s_ = 0 V) and drain (V_D_ ≤ 0 V) potentials for these 3^rd^ quadrant transistors, and increasing gate size preferentially dedoped the channel region in proximity to the drain contact, amplifying the asymmetry effect.

We further demonstrated this phenomenon specifically for high-performance transistors by fabricating two high current (large W/L aspect ratio), high transconductance transistors with equal channel size, differing only in the ratio between contact sizes (Supplementary Fig. 3A). The transistor with a 10^2^× asymmetry between its contacts exhibited three orders of magnitude improvement in lowering saturation slope compared to the symmetrical contact transistor (Supplementary Fig. 3B). The saturation region slope is a critical feature for designing voltage amplifiers because the smaller the slope, the more output voltage changes with respect to small input currents^23^. Minimizing saturation slope leads to higher achievable gain and permits efficient cascading of transistors for formation of multi-stage amplifiers. We then used the data derived from these transistors in the common-source amplifier circuit model with load resistances (R_L_) that were matched to channel resistances to maintain a balanced output voltage and calculated gain (half the supply voltage, Supplementary Fig. 3C).

Next, we used this asymmetrical design to create PEDOT:PSS-based transistors that can operate in the first-quadrant, similar to n-type devices, but are comprised only of a hole-conducting polymer. We arranged the contact asymmetry such that the lowest potential contact, which in this case was the source (V_S_ = 0 V), had the smallest area to maximize dedoping in this region (Fig. 1C, D). Systematic scaling of the contact asymmetry revealed that the first-quadrant saturation region was drastically enhanced when the source contact had a significantly smaller area (~10×) than the drain contact and continued to improve with increasing asymmetry (Supplementary Fig. 4A, B). For these 1^st^ quadrant transistors, the gate electrode area was comparable in size to the larger contact area. This configuration allows the gate and drain potentials (V_D_, V_G_ > 0) to remain higher than V_S_ and induce cation drift into the source region, leading to modulation of the drain current (Fig. 1C, D) and consistent with earlier work investigating the electrochemical electrode coupling (EEC) in the presence of a mixed conducting gate electrode^40^. To validate this theory, we varied the gate area (A_G_) relative to contact area for a 1^st^ quadrant-operating transistor with asymmetrical contacts (1:100). We determined that in order to preserve the region in proximity to the source contact as the most dedoped, the gate area should be comparable to that of the drain contact to minimize dedoping adjacent to this contact (Supplementary Fig. 5). Leveraging these results, we created high-performance 1^st^ quadrant-operating transistors with a hole-conducting channel material (PEDOT:PSS; Fig. 1E). We found that our contact asymmetry approach permitted geometrical scaling because introduction of this design element improved transistor function regardless of channel size (Fig. 1F, Supplementary Figs. 6 and 7). This property is important to enable tailoring of transistor properties for specific applications. Furthermore, our approach is not restricted to a specific material or charge carrier; it is amenable to both n- and p-type mixed conductors and can facilitate creation of complementary circuitry using a wide range of mixed-conducting materials, including PEDOT:PSS, PEDOT:PSS and polyethylenimine (PEI) blend, n-doped poly(benzodifurandione) polyethylenimine, poly(benzodifurandione) (PBDF:PEI), poly(benzodifurandione) (PBFDO), and functionalized contorted acene ribbons (CAR) (Fig. 1G, Supplementary Fig. 8).

**Fig. 2:**
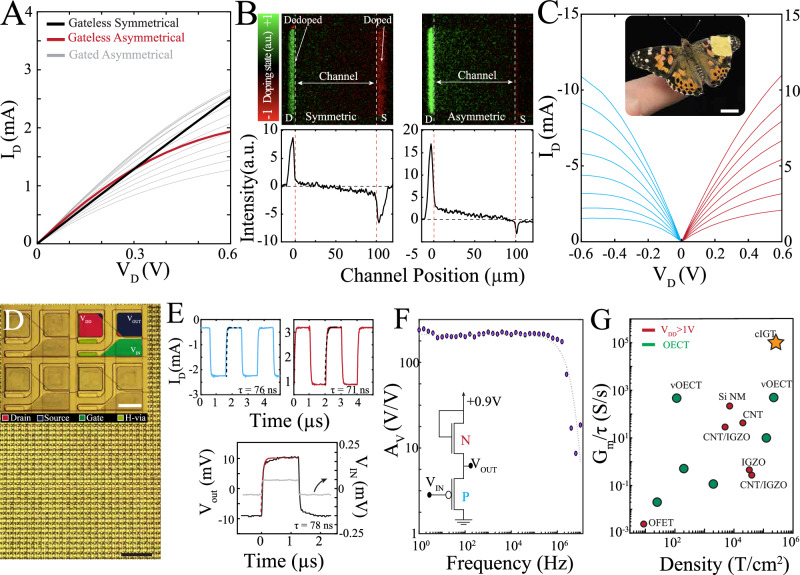
Larger contact area defines the most dedoped region of the channel leading to high-gain, densely packed conformable complementary circuits. **A** Output characteristics of an asymmetric IGT (gray). Red and black curves demonstrate I_D_ of asymmetrical and symmetrical contact area-based IGTs respectively, in the absence of a gate potential. 1^st^. **B** Optical moving-front measurement of the channel of a symmetric (left) and asymmetric (right) gateless PEDOT:PSS-based transistor. Doping-state determined by image subtraction of data generated in the unbiased condition from that acquired after application of –0.6 V bias from source to drain (W, L = 100 µm A_D_ = 100 ×100 µm^2^, A_s_ = 100 ×1500 µm^2^). Lower plots demonstrate vertical summation of pixel intensity at each horizontal channel/contact position (red dashed lines demarcate transition between contact and channel). Note the upper and lower graphs are aligned, as indicated by red and white dashed lines. **C** Output characteristics of a cIGT pair based on PEDOT:PEI blend with comparable current range. Inset: optical image of a conformable array of 129032 cIGT pairs placed on the surface of a flapping wing of a live butterfly (scale bar, 10 mm; V_G_ range = –0.1–0.6 V). **D** Optical micrograph of a densely packed cIGTs (258064 transistors/cm^2^; scale bar, 100 µm). Inset shows magnified colorized image of 4 pairs of cIGTs (scale bar, 10 µm). **E** Transient response of 3^rd^ (blue) and 1^st^ (red) quadrant operating transistors, as well as cIGT-based (black) common-source amplifier generated by square pulse input signals (top; V_G_ = 500 mV; 500 kHz, bottom: V_IN_ = 100 µV; 500 kHz). Time constants were obtained from fitting the voltage response to an exponential function (dashed lines). **F** cIGT voltage gain as a function of frequency demonstrating a uniform 46 dB (~200 V/V) gain with 2 MHz corner frequency (V_in_ = 100 µV_peak-to-peak_). **G** Comparison of complementary transistors based on the maximum transconductance and time constant for n- and p-type materials. Transistor density was calculated using the highest density of operable devices within a circuit.

How does contact asymmetry modulate dedoping and thereby transistor operation? Our geometric studies suggested that when contacts are asymmetric, the larger contact can dedope the conducting polymer in its vicinity. To further investigate this hypothesis, we fabricated pairs of transistors with equal channel geometries (100 µm × 100 µm; horizontal channel) that differed based on whether they had symmetric or asymmetric contact areas, and did not connect the gate electrodes. This set-up resulted in a “gateless” condition because the gate electrode was absent from the transistor circuit. We swept the drain potential (V_D_ = −0.6 to +0.6 V) in this gateless set-up and found that the transistor with asymmetric contacts (Fig. 2A, red trace) exhibited saturation whereas its symmetrical counterpart did not (Fig. 2A, black trace). These results indicate that the larger contact can function as a sort of gate (“pseudo-gate”) that can effectively mediate channel dedoping in proximity to the lowest potential contact. Similar outcomes were obtained for the 1^st^ and 3^rd^ quadrants by manipulating the location of the larger contact area (Supplementary Fig. 9), suggesting that the main driver of local channel dedoping is proximity to this contact^38,40,44^. To visualize this putative mechanism directly, we developed an *in operando* optical moving-front measurement set-up that allowed visualization of the doping level of each contact and the intervening transistor channel in the absence of any gate potential. When contact areas were symmetric, they exhibited a similar level of dedoping (drain contact, green) and doping (source contact, red) after application of the drain potential (V_D_ = −0.6 V; Fig. 2B, left) compared to the unbiased channel. Consequently, the doping state of the channel progresses from a dedoped state (close to drain) to equal magnitude doped state (close to source). In contrast, asymmetric contact areas established a strong area of dedoping in proximity to the drain contact (smaller contact, green) and a weaker area of doping in proximity to the source contact (larger contact, red; Fig. 2B, right). In this case, the doping state of the channel progresses from a highly dedoped state (close to drain) to a minimally doped state (close to source) leading to significantly greater overall channel dedoping and capacity for saturation (Supplementary Fig. 10).

The ability to fabricate complementary transistors with the same material is highly advantageous for creating conformable, closely packed, consistent transistors. We were able to achieve a density of 258,064 transistors/cm^2^ using vertical channel cIGTs (Fig. 2D). Of note, vertical and horizontal IGTs are similar in regard to device functionality due to the non-overlapping source and drain electrodes in both cases (Supplementary Fig. 1). This set-up is in contrast to traditional vertical channels in OFETs or OECTs where the channel material is sandwiched between two metal contacts (material thickness defines the channel length) and it is possible to introduce undesirable parasitic capacitances and leakage paths^45^. Because the mechanism of operation is consistent regardless of quadrant of operation, the degree of dedoping can be accurately controlled by tuning the potential that de/dopes the channel. For 3^rd^ quadrant operating devices, the degree of dedoping is represented by (V_G_ – V_D_) and for 1^st^ quadrant devices it is (V_G_ – V_S_). Therefore, we were able to generate 1^st^ and 3^rd^ quadrant-operating transistors with symmetrical output characteristics via geometrical tuning (Fig. 2C). This design approach maintains the high-speed operation inherent to IGTs, as channel de/doping still occurs via local ion reservoirs within the polymeric channel (Fig. 2E). Within this framework, we created voltage amplifiers with uniform 200× gain and greater than 2 MHz effective bandwidth (Fig. 2F, Supplementary Fig. 11). Overall, cIGTs enable development of conformable complementary circuits and amplifiers with gain-bandwidth and density characteristics that compare favorably to currently available transistors due to their scalable fabrication, ability to establish symmetrical currents, and compatibility with a wide range of high performance mixed-conductors (Fig. 2G, Supplementary Fig. 12, Supplementary Tables 1 and 2).

The ability to amplify electrophysiologic signals in the immediate vicinity of their source can substantially improve sensitivity, facilitating detection of weak activity patterns that would otherwise be obscured by noise^46–49^. We previously demonstrated the advantage of a current amplifier in acquiring signals from the brain^13,20^. However, current amplifiers are difficult to integrate with existing voltage data acquisition systems, and they require an additional voltage amplifier stage to scale the signal for efficient use of the dynamic range of the analog to digital converter. Moreover, current amplifiers are more susceptible to noise when long interconnects need to be employed in a device because small resistance changes in the interconnects, which are carrying high-current output, could add unwanted voltage bias.

**Fig. 3:**
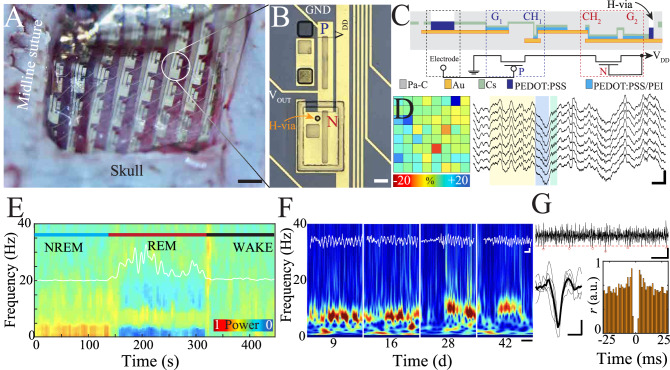
Conformable cIGT-based amplifier array for stable and chronic in vivo electrophysiological recordings at the resolution of individual action potentials. **A** Intra-operative micrograph of cIGT-based amplifiers conforming on the pial surface of rat brain (scale bar, 500 µm). **B** Optical micrograph of a cIGT-based amplifier consisting of active and load transistors (scale bar, 50 µm). **C** Cross-sectional schematic of cIGT-based voltage amplifier and its corresponding circuit. **D** Deviation of individual amplifier gain from mean (left). Raw LFP time traces of posterior parietal cortex with visible sleep spindle (yellow) and cortical ripple oscillatory activity (blue: delta wave; green: cortical ripple; yellow: sleep spindle, scale bar, 200 ms, 500 µV). **E** Time frequency spectrogram of cortical recording highlighting transition from NREM to REM and wakefulness. The white superimposed trace is the ratio of delta (0.5–4 Hz) to theta (5–8 Hz) oscillations as a parameter used for automatic state differentiation. **F** Longitudinal recording of sleep spindles highlighting consistent, stable power and frequency content of cIGT-based neural acquisition over a 40-day period (white scale bar, 200 ms, 100 µV; black scale, bar 1 s). **G** Individual action potentials (location denoted by red circles) detected using a band pass filter (250−2500 Hz) and amplitude thresholding (dashed red line; top; scale bar, 1 ms, 30 µV). Overlay of multiple detected individual action potential waveforms with average waveform in bold (lower left, scale bar, 1 ms, 30 µV). Auto-correlation of spike times for a sample putative single neuron acquired by cIGT-based amplifiers demonstrating physiologic refractory period.

In parallel to these circuit design properties, amplifiers targeted at interfacing with delicate tissue, such as the brain, should be biocompatible, conformable, and capable of stable long-term recordings. Because cIGTs meet both these circuit and biologic criteria, we designed and fabricated an array (8 × 8; 450 µm spacing) of bipolar amplifiers based on cIGTs (Fig. 3A, Supplementary Fig. 13). We utilized a first-quadrant operating device (similar to an n-type transistor) to create a constant current source. This current source was used to establish the drain current of a 3^rd^ quadrant operating transistor, biased at its saturation region to optimally function as an amplifier (Fig. 3B, C).

To test the functionality of the devices, we performed long-term in vivo electrophysiologic recording in freely moving rodents. The conformable cIGT transistor array was placed on the pial surface of rat posterior parietal cortex. The cranial window was covered with gel-foam and a biocompatible gel to complete the implantation (Fig. 3A). Powering and data digitization were accomplished using a custom head-stage compatible with existing neurophysiological data acquisition systems (Methods). After recovery from surgery, we recorded neural activity from these cIGT-implanted rats during natural behavior for more than 40 days. To validate the physiological nature of the data generated and investigate device long-term reliability, we analyzed its spectral and oscillatory content. We identified epochs of high delta (0.5–4 Hz) power in the local field potential (LFP) consistent with non-rapid eye movement (NREM) sleep, high theta (5–8 Hz) power indicative of REM sleep, and desynchronized waking activity (Fig. 3D, Supplementary Fig. 14). Data quality was sufficient to enable automated state classification with an established protocol (Fig. 3E)^50,51^. Next, we focused on activity patterns characteristic of NREM sleep, a brain state critical for establishing brain synchrony^52^. We identified thalamocortical spindles and cortical ripples, key oscillations involved in memory consolidation,^53–55^ in each day of cIGT-acquired data. Spectral features of detected spindles were consistent over time, emphasizing the device stability (Fig. 3F).

The ability to detect and isolate action potentials attributable to individual neurons is critical for interpretation of neural data. This analytic process requires a sampling rate of 20–30 kHz to sufficiently characterize action potential waveforms. Because our cIGT-based amplifiers have a time constant (~%63 of fully charged state) of approximately 75 ns, they surpass the requisite 10–30 µs time constant necessary to accurately capture the action potential waveform. Therefore, we evaluated the cIGT-acquired data for this high spatiotemporal resolution activity. We band-pass filtered the data (250–2500 Hz) and were able to detect individual extracellular action potentials from the surface of the brain (Fig. 3G; top). These spikes could be clustered to identify putative single-unit activity with physiologic refractory periods (Fig. 3G; bottom). These results indicate that cIGTs can be fully implanted in freely moving rodents to provide stable, high amplification of neural data at the interface with the brain.

We hypothesized that the properties of cIGTs and their demonstrated stability for long-term amplification of neural signals would enable us to deploy this technology in experimental paradigms in which current devices are unsuitable and prohibit neural data acquisition. One such paradigm, which parallels many clinical applications, is the developing brain, where lack of conformable, miniaturized, and fully implantable neural amplifiers provides major challenges to understanding cortical network maturation. To acquire physiologically relevant signals, the organism should be monitored longitudinally in naturalistic environments that permit maternal care and social interactions^43,56^. However, conventional approaches for high spatiotemporal monitoring require implantation of bulky, rigid components that cause tissue disruption in a growing animal^56–59^. Current state-of-the-art neural acquisition systems are at the boundary of acceptable weight-based limits for use in adult mice, and are therefore prohibitively heavy/large for mouse pups^60–64^. Their extruding components can also interfere with the necessary maternal care, negatively impacting neurodevelopment^65,66^. We thus aimed to create a fully implantable cIGT-based neural interface device for this application. Fully implantable devices require high voltage-gain amplification not only to amplify weak physiological signals, but also to eliminate the need for any rigid amplifiers in the following stages of the signal chain. Critically, this high-gain operation should be performed with supply potentials less than that of water hydrolysis (1.2 V) to eliminate risk of tissue damage and unwanted electrical stimulation. Practically, it has been found that gains in the range of 50–200× are needed for electrophysiological signals (10 µV–10 mV)^13,28,67^. This range of gain ensures sufficient amplification of the signals while maintaining the dynamic range of the signal voltage within the boundaries required for most analog to digital converters. A symmetrical IGT transistor^14,20^ can be utilized as an effective current amplifier to buffer weak physiological signals, but this approach does not provide the required voltage amplification. To create a voltage amplifier, the IGT can be deployed in a common-source voltage amplifier configuration in conjunction with a load resistor (R_L_). The higher the R_L_, the larger the gain (I_D_ × R_L_). However, R_L_ also proportionally increases the supply (V_DD_) voltage. A symmetrical IGT-based, resistor load common-source amplifier with <1.2 V supply voltage does not have sufficient gain for a fully implantable device (Supplementary Fig. 15). Asymmetrical contact areas enhance the saturation region of any given IGT, which improves the voltage-gain by increasing the effective output resistance (R_OUT_) through enlarging channel resistance (r_0_; Supplementary Fig. 16A). However, even an asymmetrical IGT-based common-source amplifier with resistor load cannot provide sufficient gain at <1.2 V supply voltages. To further improve the gain, the load resistor can be replaced by a constant current source. The combination of the constant drain current and the small slope saturation region translates small input changes into very large voltage changes (Supplementary Fig. 3C). For a 3^rd^ quadrant operating IGT, this constant current-source can only be established by a 1st quadrant operating transistor (positive gate and drain potentials) biased at the saturation region by connecting its gate and drain together (Supplementary Fig. 16B). Therefore, it is necessary to have complementary IGTs to achieve the required voltage gain at low supply voltages (Supplementary Fig. 16C–E).

**Fig. 4:**
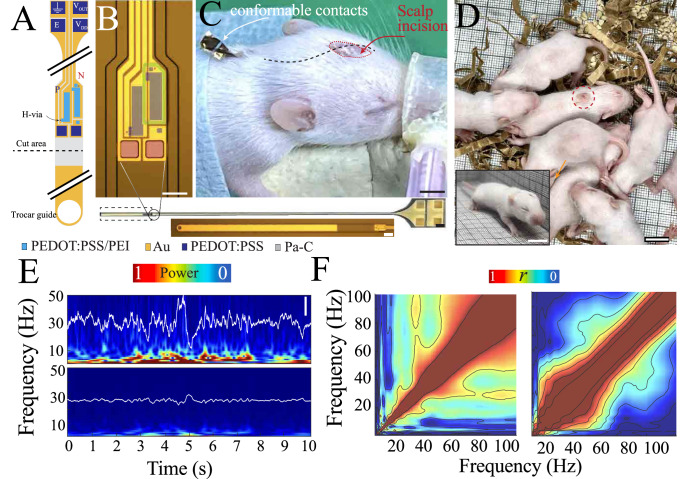
Fully implantable cIGT-based amplifier for acquisition of neural activity over the course of brain development. **A** Schematic of the fully implantable cIGT-based probe with axis breaks showing the electrical contacts for recording (top), the cIGT amplifier (middle), and the anchor hole (bottom). **B** Optical micrograph of a cIGT-based front-end amplifier (top; scale bar, 80 µm) located at the tip of the implant (middle; scale bar, 1 mm). Optical micrograph of the trocar-guided section of the device with Au-coated strip to indicate location for detachment (bottom; W, L = 20, 2 µm; scale bar, 100 µm). **C** Post-surgical image of implanted pup (P9) with red circle highlighting the closed scalp incision. Dashed black line indicates subcutaneous location of the device interconnects, attaching to conformable contacts externalized for recording (scale bar, 3 mm). **D** Image of pup implanted with cIGT-based front-end amplifier (implantation site indicated by dashed red line) surrounded by littermates in a naturalistic setting (scale bar, 10 mm). Inset shows the same mouse pup at P12 after having acquired ambulation as a natural developmental milestone (scale bar, 10 mm). **E** Comparison of LFP time traces (white) and their corresponding time-frequency spectrogram from mouse pup at age P14 that was implanted at P9. The traces and the heat map are scaled based on noise-level (scale bar, 300 µV). **F** Comparison of cross-frequency coupling of data acquired by chronically implanted cIGT vs. conducting polymer electrode. CIGT data reveals coupling between the spindle band and gamma band activity that is obscured in the conducting polymer electrode data.

To test the capacity of cIGTs to acquire longitudinal neurophysiologic data from developing mouse pups, we created conformable cIGT-based amplifiers and interconnects. Device conformability enabled i) use of a trocar needle for minimally invasive implantation; ii) a miniaturized craniotomy; iii) a significantly simplified surgical procedure; and iv) closure of all incision and wounds with no extruding components. Ohmic contacts for data transmission were placed in the animal’s dorsal subcutaneous tissue to take advantage of larger available surface area in this region compared to the scalp. In such a set-up, which is necessary due to the small size of the animal, interconnects are in close proximity to musculature. Therefore, signal amplification must occur prior to these interconnects and the output impedance of the amplifiers should be significantly lower than the next stage to minimize any noise contamination from movement or electromyographic activity. We designed a 36 mm long probe that can increase in length by 4 times via unfolding to accommodate animal growth across development. The tip of the probe was equipped with a small through-hole (80 µm) that enabled temporary attachment to a 100 µm stainless steel stylet which serves as a trocar to guide the probe into position (Fig. 4A, B). We designed the interface of the probe to consist of both a conducting polymer-based electrode and a cIGT-based amplifier to permit comparison of data acquired by these components. Once the probe was positioned above the implantation site, the trocar-anchoring segment was cut using surgical scissors to further minimize the physical footprint of the implant. After placement of the electrode and amplifier on the surface of the brain, interconnects routed the acquired data through subcutaneous tissue to implantable in-body contacts. Electrical ohmic connection between the implanted device and the external electronics was established using mixed-conducting particulate composites (MCP^38^) which can be removed and reapplied several times to facilitate multiple epochs of recording.

We implanted these conformable probes in mouse pups at postnatal day 9 (P9). The simplicity of the device and connections allowed surgery to be completed in 30 minutes, improving surgical outcome and decreasing potential for adverse effects (Supplementary Fig. 17). Daily data acquisition was performed by placing the pup in an incubated recording box that mimics the environment of the nest and connecting the implanted device to external electronics. The pup was not restrained by this flexible connection and was able to move freely. After the completion of each recording session, pups were returned to their litter to receive maternal care (Fig. 4C). The ability to return the pup to its mother and littermates is critical to ensure normal development and physiologic relevance of acquired data. We analyzed the data acquired by the device from each day of development (P9-P14). We observed cortical oscillatory patterns characteristic for these developmental ages, including spindle- and gamma-band activity. To ensure that these data were not contaminated by muscle activity, we performed concurrent electromyography (EMG) using a temporarily implanted needle electrode and confirmed a lack of coherence between the signals derived from muscle and brain (Supplementary Fig. 18A). Next, we the compared data generated by the conducting polymer-based electrode and cIGT-based amplifier. The electrode and amplifier captured signals that were coherent in the low frequency domain, as expected due to their close proximity to each other, and thus their putative interface with similar neural populations. However, the cIGT-based amplifier data was both higher amplitude and exhibited enhanced spectral content in the higher frequency bands (Fig. 4E, Supplementary Fig. 18B). Cross-frequency coupling of the neural data acquired by the cIGT-based amplifiers, but not the conducting polymer-based electrode, demonstrated clear frequency co-modulation and nesting of higher frequencies in the period of slower oscillations (Fig. 4F, Supplementary Fig. 18C). These results suggest that cIGT-based amplifiers provide high spatiotemporal resolution data when fully and chronically implanted in freely moving organisms, creating an improved signal to noise ratio compared to passive conducting polymer-based electrodes and enabling experimentation that is currently impractical with Si-based rigid electronics.

## Discussion

Organic electronic devices are desirable for biologic applications because they offer enhanced biocompatibility and conformability. However, ability to integrate such devices into high performance functional circuits performance lags Si-based electronics, with a key limitation being lack of matched, complementary transistors that can be fabricated from a single material. Here, we discovered that manipulation of contact asymmetry in internal ion-gated organic electrochemical transistors allows tuning of the saturation regime and creation of n- and p-type transistors using the same conducting polymer channel material (cIGTs). We furthermore determined the mechanism underlying this phenomenon using gate-less electrical measurements and *in operando* optical moving-front experiments to visualize channel dedoping. We revealed that the contact with the largest area acts as a pseudo-gate, capable of creating a localized region of the channel with maximal de/doping which governs the saturation slope. The size of the lowest potential contact relative to the other contact then determines the transistor’s quadrant of operation.

This phenomenon is strongly tied to the geometry of all transistor components. In order for the contacts to be capable of drifting ions within the channel, their area must exceed that of the channel. This aspect ratio is prevalent in transistors with vertical channels, for instance when interlayer thickness is used to determine channel size but contacts are patterned with optical lithography. Gate size must also be chosen relative to contact size and desired quadrant of operation. For most effective 3^rd^ quadrant operation, gate size can be maximized because the stronger applied potential amplifies the effects of contact asymmetry. In contrast, gate size cannot be increased beyond that of the largest contact (drain) for efficacious 1^st^ quadrant operation because doing so would decrease the potency of local dedoping adjacent to the smaller contact (source).

We found that cIGTs created according to these design rules are high performance, with transconductance and time constants that surpass flexible n- and p-type transistors fabricated with multiple different, often non-biocompatible, materials and solvents. Their output characteristics can also be precisely matched to enable seamless creation of integrated circuits. Because a single material is used, with geometrical manipulation sufficient to dictate operating characteristics, the fabrication process for closely packed transistors with a small physical footprint is substantially simplified compared to current approaches. These properties are ideal for devices that require local amplification and processing of potentially low amplitude and weak biological signals that co-exist with multiple sources of electrical noise, such as is necessary in pacemakers, responsive neurostimulation devices, and brain machine interfaces^68–70^. Furthermore, cIGTs maintain superior processing capacity in a fully conformable state, permitting use in fragile tissue. Specifically, we showcase these features by demonstrating effective chronic use in a developing organism, where tissue is not only sensitive, but expanding as the animal grows. Although conducting polymer electrodes are capable of high-quality acute recording in head-fixed developing animals^56^, cIGT-based amplifiers demonstrated enhanced performance when employed in chronic, freely moving settings. In keeping with previous characterization of IGTs^14,20,22^, cIGTs were also found to be stable in a physiologic environment for an extended period of time and enabled tracking of neural activity patterns across development. Such longitudinal monitoring is increasingly critical for bioelectronic devices, as it is established that diagnostic and therapeutic interventions require adjustment over time to personalize and optimize outcomes.

Overall, we have demonstrated that organic electronics can be tuned to create robust complementary integrated circuits capable of high-quality acquisition and processing of biological signals. cIGTs and their foundational principles offer the possibility of broadening application of organic electronics to devices that conventionally rely upon bulky, non-biocompatible components.

## Methods

### Material preparation

All chemicals were acquired from Sigma Aldrich unless otherwise stated. Pa-C dimer was acquired from Specialty Coating Systems for chemical vapor deposition. AZ nLOF 2020 and AZ 10XT photoresists as well as AZ400K and AZ 300 MIF developers were purchased from MicroChemicals. PEDOT:PSS aqueous dispersion (Clevios PH1000 from Heraeus) was mixed with D-Sorbitol (40% w/v), 4-dodecylbenzene sulfonic acid (1% v/v), and 3-Glycidyloxypropyl-trimethoxysilane (1% v/v) to form a solution processable transistor channel material. PEI was diluted in DI water (1% v/v) to create a solution processable dedoping solution. Chitosan was diluted with acetic acid (6% w/v) to form a solution processable ion membrane. Micro-90 alkaline cleaning solution (from Special Coating Services) was diluted with DI water 5% and 0.3 v/v as anti-adhesion layers for Pa-C and Si, respectively.

### Device fabrication

100 mm diameter Si wafers (single side polish, 550 µm thickness) were spin coated at 1500 rpm for 30 s with a 0.3% soap anti-adhesion layer. 2 µm of Pa-C was consequently deposited through chemical vapor deposition (CVD) using a SCS Labcoater 2. The first metal layer pattern was created using nLOF 2020 negative photo resist using standard optical photolithography. Negative resist was spin coated at 3000 rpm for 30 s and then baked at 110 °C for 90 s. Resist was exposed using a chrome photomask on a Suss MA6 mask aligner. Devices were baked for 90 s at 110 °C after exposure and developed in AZ 300 MIF. The Angstrom EvoVac Multiprocess evaporator was used to deposit 10 nm of Ti and 150 nm of Au through electron-beam evaporation. The metal was patterned with a lift off technique assisted by sonication in a 1 L acetone bath for 20 min. A second Pa-C film of 50 nm was deposited through CVD, with the assistance of a secondary diffusion barrier chamber (aperture 1 mm). A second metal layer was deposited with similar parameters as layer 1. A 2 µm Pa-C film was then deposited with adhesion promoter and followed by spin coating an 5% soap antiadhesion layer at 1500 rpm for 30 s. Next, an additional 2 µm Pa-C was deposited to act as a sacrificial layer for patterning. The AZ 10XT positive photoresist was spin coated at 300 rpm for 10 s and then 5000 rpm for 30 s before baking at 110 °C for 90 s. Resist was exposed using a chrome/soda lime mask and developed using two 20% v/v diluted baths of AZ 400 K in DI water for 3.5 minutes each. Pa-C was etched using an oxygen plasma reactive ion etching process (Oxford Plasmalab 80; 180 W, 50 s.c.c.m. O_2_ and 2 s.c.c.m. SF_6_) for 14 min. The prepared PEDOT:PSS transistor channel material was spin coated onto the devices at 5000 rpm for 30 s and baked at 110 °C before being patterned by peeling off the sacrificial layer. PEI solution was spin coated at 1500 rpm for 30 s and followed by a 120 s bake at 110 °C for enhancement mode devices. Prepared chitosan solution was spin coated at 1500 rpm for 30 s and baked at 110 °C for 5 min to create an ion membrane. A 400 nm layer of Pa-C was deposited with the adhesion promoter to allow for patterning of the ion membrane. The positive photoresist and oxygen etching process was repeated to pattern the ion membrane. An additional 2 µm Pa-C film with adhesion promotor was deposited and followed by spin coating an antiadhesion layer of 5% v/v soap. Another sacrificial 2 µm Pa-C film was deposited with the same CVD process. The negative photoresist process was repeated to pattern 50 nm of Ti as a hard etch-mask to pattern the electrodes and hydration vias, which utilize the Au layers as etching stops, as well as the outlines of the probes, where the Si substrate serves as an etching stop. The devices were spin coated with the prepared PEDOT:PSS solution and the sacrificial layer was peeled off to pattern the polymer. Devices were then peeled from the Si substrate with the assistance of DI water.

### Electrical characterization

The Keysight B2902A precision source measurement unit (SMU) and Keysight BenchVue 2019 and B2900 Quick IV software were used to acquire current voltage measurements of transistors.

### Optical characterization

The Keysight B2902A precision source measurement unit (SMU) was used to supply a constant voltage to horizontal transistors with no gate electrode. Optical imaging was performed with an Amscope FMA050 microscope camera attached to an Amscope microscope set to 5x magnification. Images were taken with no V_D_ bias and with –0.6 V bias before being converted to greyscale and subtracted to result in a differential image which was recolored by intensity.

### Temporal characterization

Temporal responses were recorded by the Keysight InfiniiVision EDUX1002A oscilloscope with gate voltage pulses supplied the Keysight 33500B series function generator and a drain voltage supplied by an SMU. Data was fitted using Origin 2024b.

### Gain characterization

The gain measurements utilized the aforementioned function generator in conjunction with a voltage divider to provide a 100 µV input signal to the gate. A 1 V power supply was provided by a SMU and the output was recorded with the Micsig STO1104C oscilloscope in AC coupling mode by averaging 128 waveforms to reduce noise. For each data point, we supplied a 100 µV peak-to-peak sinusoidal wave using a function generator as small-signal input and measured the peak-to-peak amplitude of amplifier’s V_out_ using embedded functions of an oscilloscope for at least 10 cycles. This approach provided the flexibility to tune the measurement time per frequency to obtain multiple cycles and improve the accuracy of the measurement.

### Animal surgical procedure

All animal experiments were approved by the Institutional Animal Care and Use Committee at Columbia University. Male Long–Evans rats (380 g; n = 3) were used for intracranial implantation and chronic recording. Swiss-Webster mouse pups were implanted on postnatal day (P) 9 and recorded through P14 (n = 3). All signals were acquired via an RHD2164 die (Intan Technologies) recording at 20,000 samples/s utilizing a custom printed circuit board. Signals were visualized in real‐time with the RHD2000 Interface Software. Data was analyzed in MATLAB (MathWorks). Because electrophysiological signals range between 20 µV – 1 mV, the gain of cIGT amplifiers was adjusted by the means of channel geometry to maintain the output voltage within the range of the acquisition system’s analog to digital converter (±5 mV).

### Chronic rat implantation

The rats were initially anaesthetized with 2.0% isoflurane and maintained under anesthesia with 0.75–1.0% isoflurane during the surgery. To minimize brain swelling and inflammation, methylprednisolone (30 mg kg^–1^) was administered during surgery. A 3 × 3 mm^2^ cranial window over the posterior parietal cortex (AP, −3.5 mm; ML, 3.5 mm) was opened and the dura mater was removed. The cIGT probe was placed on the cortex and the craniotomy was covered with gel-foam, then sealed using a silicone elastomer. Chronic recordings were performed in two-hour sessions and no other experimentation occurred with the same animal.

### Chronic mouse implantation

Swiss-Webster mouse pups (5–10 g, 8–13 days of age, male) received acute and chronic device implantation and electrophysiologic recording. Anesthesia was induced with 3.0% isoflurane and maintained with 1.0–1.5% isoflurane. Minimized time under anesthesia expedited postoperative recovery. A small skin incision and a burr hole craniotomy (centered at AP −1.5 to −2 mm, ML 1–1.5 mm) were made for cortical device implantation. Another set of incision and burr hole was made above the cerebellum for reference. A 23-gauge needle was used to pierce into the nuchal subcutaneous space and advanced to emerge above the exposed cortex, acting as a temporary catheter for flexible device placement. An acupuncture needle (100 µm diameter) was attached to the anchor at the tip of the flexible device with glue, and used to maneuver the interconnect ribbon through the catheter. The anchor at the tip was then trimmed to detach the acupuncture needle, and the catheter was removed, leaving only the connection pads exposed. The electrodes were advanced through the cortical burr hole to conform to the dorsal cortical surface. The burr hole was covered with gel-foam and the incision site was closed with veterinary glue. For acute recording, mixed conducting particulate composite (MCP) was used to create stable electrical interfacing between the flexible connection pads and preamplification circuitry. The pup was transferred into a custom recording chamber for anesthesia recovery and signal acquisition. After recording, connection pads were detached from the preamplifier using deionized water. The pads were then tucked into the subcutaneous space. Pups were continuously monitored for 30 min before being returned to their home cage. For subsequent days of recording, mouse pups were anesthetized briefly to allow access to the contact pads and connection with preamplifier. They then underwent recording in the custom chamber, followed by detachment of contact pads and return of the animal to its mother and litter.

### Neurophysiological signal acquisition and preprocessing

A custom-made head-stage printed circuit board (EuroCircuits) was directly attached to the cIGT-based amplifiers to digitize the signals at 20 kHz for storage and offline analysis in 16-bit format. MATLAB (MathWorks) was used for data analysis and Neuroscope enable data visualization. Local field potentials were generated by down-sampling to 1250 Hz. We detected epochs of quiescence via the motion signal of the accelerometer attached to the head-stage and absence of electromyogram (EMG) artifacts. NREM sleep epochs were then identified by locating periods of elevated delta oscillations (0.5–4 Hz). Rapid eye movement sleep (REM) epochs were distinguished by an increased theta–delta-band frequency ratio. This approach to sleep scoring has been previously validated and was performed via custom MATLAB code (The State Editor^71^).

### Neural spike detection

Extracellular neural spiking was detected from each channel. Raw recordings were band-pass filtered at 250–2500 Hz. Noise floor was defined as 3 times the standard deviation of 10 s of band-pass filtered signal in NREM sleep. Negative peaks greater than this noise floor were detected as spikes. Co-occurring spikes across more than eight channels were deemed artifactual and removed. The detected spikes were sorted using a previously established and validated template matching-based clustering algorithm (Kilosort)^72^.

### Time-frequency analysis

Analytic Gabor wavelet transform was used to obtain time-frequency spectrograms of neural oscillations. Comodulograms were computed Gabor wavelet, followed by a Gaussian window convolution with duration matched to the frequency. The auto-correlation was computed using the magnitude of the wavelet transform. Comparisons with *p* < 0.05 after Bonferroni-Holm correction for multiple comparisons were considered statistically significant.

### Reporting summary

Further information on research design is available in the Nature Portfolio Reporting Summary linked to this article.

## Supplementary information

Supplementary Information

Reporting Summary

Solar Cell Reporting Summary

Lasing Reporting Summary

Transparent Peer Review file

## Source data

Source Data

## Data Availability

All data needed to evaluate the conclusions in the paper are present in the paper and/or the Supplementary Information. All source files and experimental data are freely and publicly available at Mendeley repository with DOI of.17632/hy3xw7j2r5.1. Additional data related to this paper may be requested from the authors. Source data are provided with this paper.
