# Supplementary material for: Spatial control of doping in conducting polymers enables complementary, conformable, implantable internal ion-gated organic electrochemical transistors
Source: Nat Commun. 2025 Jan 9;16:517. doi: 10.1038/s41467-024-55284-w (PMC11717955; doi:10.1038/s41467-024-55284-w)
Supplement: Supplementary file 1 — Supplementary Information [file 41467_2024_55284_MOESM1_ESM.pdf]

Spatial control of doping in conducting polymers enables complementary, conformable, implantable internal ion-gated organic electrochemical transistors

Duncan J. Wisniewski[1,2], Liang Ma[3], Onni J Rauhala[2], Claudia Cea[2], Zifang Zhao[2], Alexander Ranschaert[2], Jennifer N. Gelinas*[3,4,5,6], Dion Khodagholy*[1,2]

1. Department of Electrical Engineering, University of California, Irvine, CA 92697, USA
2. Department of Electrical Engineering, Columbia University, New York, NY 10027, USA
3. Department of Biomedical Engineering, Columbia University, New York, NY 10027, USA
4. Department of Neurology, Columbia University Medical Center, New York, NY 10032, USA
5. Department of Anatomy and Neurobiology, University of California, Irvine, CA 92697, USA
6. Department of Pediatrics, University of California, Irvine, CA 92697, USA

* Corresponding author:
Jennifer Gelinas: gelinasj@hs.uci.edu
Dion Khodagholy: dion.kh@uci.edu

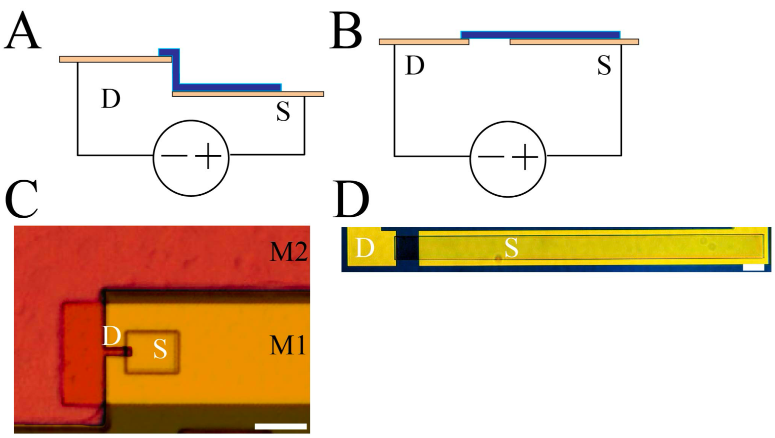

Supplementary Figure 1: Asymmetrical contact area IGTs.
A) Diagram of asymmetrical vertical transistor layout.
B) Diagram of asymmetrical horizontal transistor layout.
C) Optical micrograph of a vertical channel IGT (scale bar, 10 μm).
D) Optical micrograph of a horizontal channel IGT (scale bar, 100 μm).

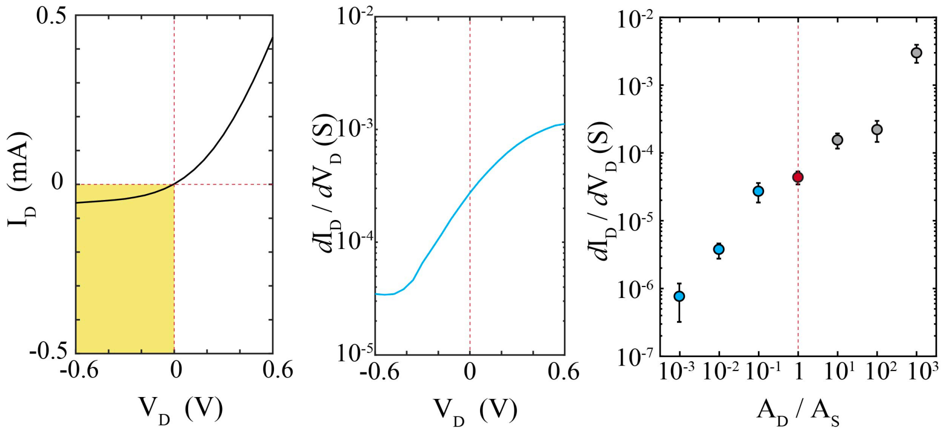

Supplementary Figure 2: Asymmetrical contact area enhances saturation region of IGTs.

A) I_D as function of V_D of the transistor in **Figure 1B** (A_S/A_D =250, W, L = 100 µm, A_G = 250k µm^2).

B) Slope of I_D as function of V_D, thereby evaluating the saturation region of the transistor at V_G = 0.6 V (A_S/A_D = 250, W, L = 100 µm, A_G = 250k µm^2).

C) The effect of contact area asymmetry on the saturation region slope at V_G = 0.6V and V_D = – 0.6 V (W, L = 100, 10 µm, n = 10 transistors per asymmetry ratio). A_D/A_S = 10^{-3}, dI/dV = $7.54 \times 10^{-7} \pm 4.33 \times 10^{-7}$ S, A_D/A_S = 10^{-2}, dI/dV = $3.69 \times 10^{-6} \pm 9.33 \times 10^{-7}$ S, A_D/A_S = 10^{-1}, dI/dV = $2.73 \times 10^{-5} \pm 8.74 \times 10^{-6}$ S, A_D/A_S = 10^{0}, dI/dV = $4.39 \times 10^{-5} \pm 9.43 \times 10^{-6}$ S, A_D/A_S = 10^{1}, dI/dV = $1.55 \times 10^{-4} \pm 3.92 \times 10^{-5}$ S, A_D/A_S = 10^{2}, dI/dV = $2.21 \times 10^{-4} \pm 7.63 \times 10^{-5}$ S, A_D/A_S = 10^{3}, dI/dV = $3.00 \times 10^{-3} \pm 9.05 \times 10^{-4}$ S.

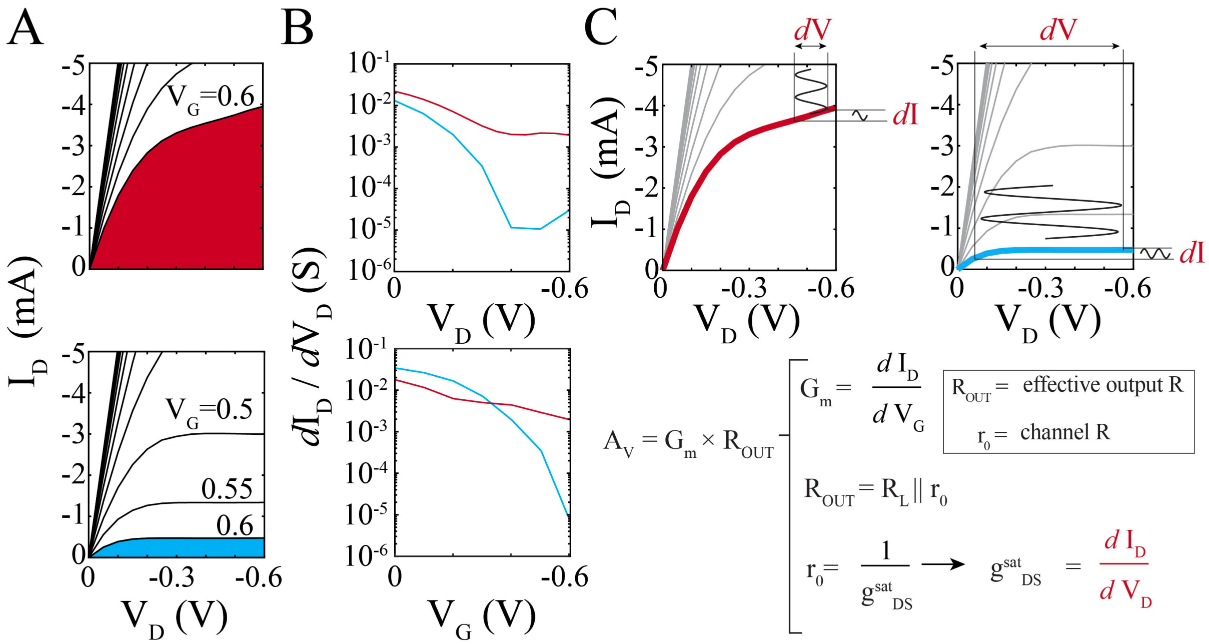

Supplementary Figure 3: Voltage gain of common-source amplifier is a function of saturation region slope.

A) Comparison of output characteristics of symmetrical (red) and asymmetrical (blue) contact area devices with similar channel geometry (A_S / A_D = 100, W, L = 500, 10 µm).

B) Asymmetrical contact area significantly reduces the saturation slope of the devices with similar channel geometry; same devices and color code as (A).

C) Illustration of the effect of saturation slope on voltage gain; blue curve with enhanced saturation yields higher voltage changes (dV) with small input current changes (dI) (top). Relationship of the voltage-gain (A_V) of a common source amplifier with transconductance (G_m) and effective output resistance. Slope of saturation (g^{Sat}_{DS}) affects the effective output resistance.

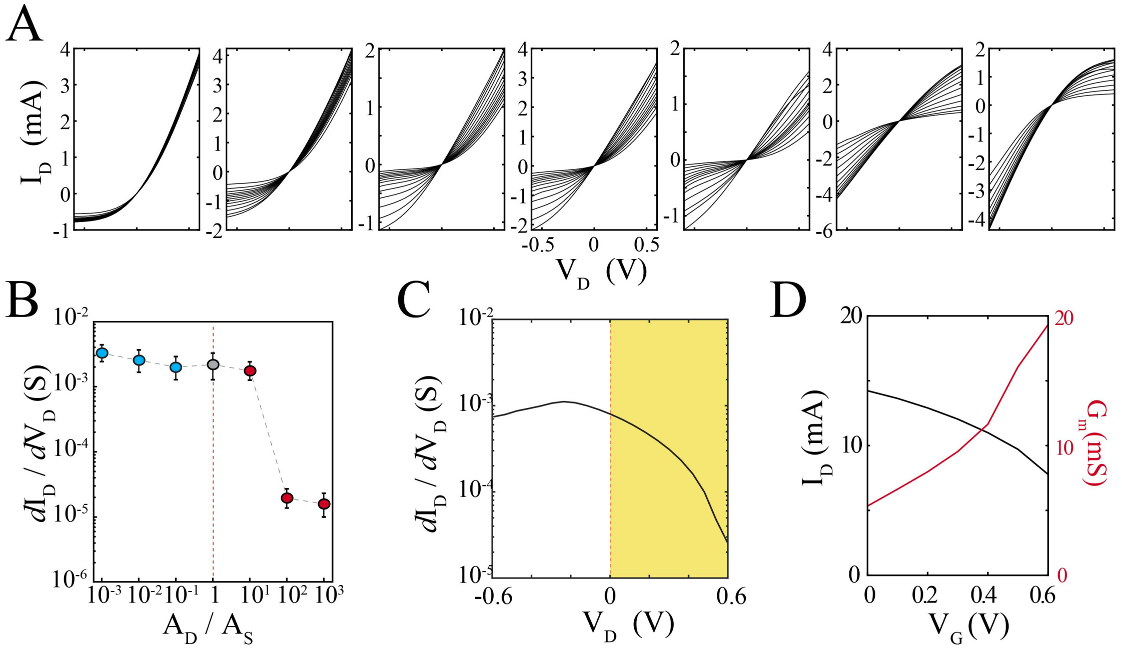

Supplementary Figure 4: Asymmetrical contact area modulates the saturation region.

A) Output characteristics of an array of devices highlighting emergence of saturation region in 1st quadrant due to asymmetry of the contacts (W, L = 100, 10 µm, and G_A = 2500 µm^2, V_D, V_G = – 0.6 V to 0.6 V, channel material: PEDOT:PSS/PEI).

B) The effect of contact area asymmetry on the slope of saturation region in the 1st quadrant (W, L = 100, 10 µm, A_G = 2500 µm^2, n = 10 transistors per asymmetry ration, V_G = 0.6 V and V_D = 0.6 V). $A_D/A_S = 10^{-3}$, $dI/dV = 3.32 \times 10^{-3} \pm 9.80 \times 10^{-4}$ S, $A_D/A_S = 10^{-2}$, $dI/dV = 2.58 \times 10^{-3} \pm 9.93 \times 10^{-4}$ S, $A_D/A_S = 10^{-1}$, $dI/dV = 2.01 \times 10^{-3} \pm 7.98 \times 10^{-4}$ S, $A_D/A_S = 10^{0}$, $dI/dV = 2.20 \times 10^{-3} \pm 9.94 \times 10^{-4}$ S, $A_D/A_S = 10^{1}$, $dI/dV = 1.76 \times 10^{-3} \pm 5.66 \times 10^{-4}$ S, $A_D/A_S = 10^{2}$, $dI/dV = 1.71 \times 10^{-5} \pm 5.75 \times 10^{-6}$ S, $A_D/A_S = 10^{3}$, $dI/dV = 1.38 \times 10^{-5} \pm 5.62 \times 10^{-6}$ S.

C) Slope of saturation of the device shown in **Figure 1E.**

D) Transfer curve (black) and transconductance (red) of the device shown in **Figure 1E** at V_D = -0.6 V.

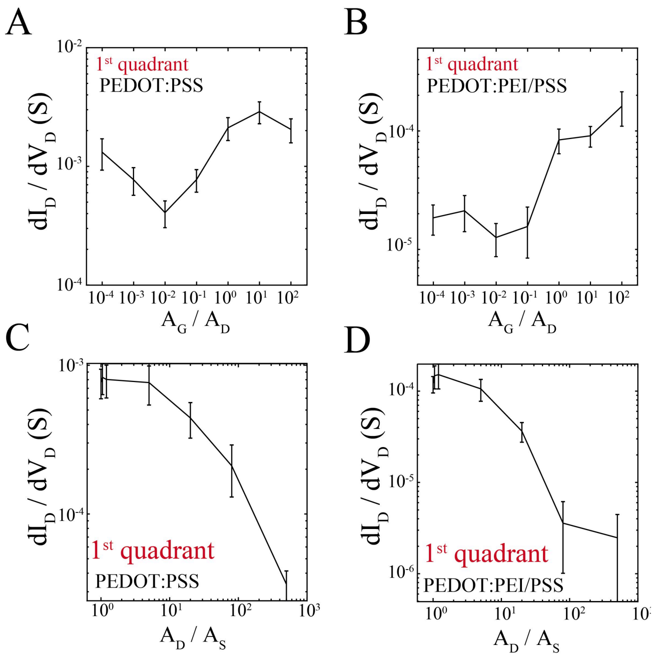

Supplementary Figure 5: Effect of gate area (A_G) and contact asymmetry on saturation slope.

A) Slope of saturation as a function of gate/channel area and the gate area/drain contact area (W, L = 100, 10 µm, A_S/A_D = 1/100, channel composition = PEDOT:PSS). V_G = 0.6 V and V_D = 0.6 V). $A_G/A_D = 10^{-4}$, $dI/dV = 1.32 \times 10^{-3} \pm 3.90 \times 10^{-4}$ S, $A_G/A_D = 10^{-3}$, $dI/dV = 7.73 \times 10^{-4} \pm 2.03 \times 10^{-4}$ S, $A_G/A_D = 10^{-2}$, $dI/dV = 4.08 \times 10^{-4} \pm 1.04 \times 10^{-4}$ S, $A_G/A_D = 10^{-1}$, $dI/dV = 7.73 \times 10^{-4} \pm 1.66 \times 10^{-4}$ S, $A_G/A_D = 10^{0}$, $dI/dV = 2.11 \times 10^{-3} \pm 4.61 \times 10^{-4}$ S, $A_G/A_D = 10^{1}$, $dI/dV = 2.89 \times 10^{-5} \pm 6.06 \times 10^{-4}$ S, $A_G/A_D = 10^{2}$, $dI/dV = 2.05 \times 10^{-5} \pm 4.68 \times 10^{-4}$ S.

B) Slope of saturation as a function of gate/channel area and the gate area/drain contact area (W, L = 100, 10 µm, A_S/A_D = 1/100, channel composition = PEDOT:PEI). V_G = 0.6 V and V_D = 0.6 V). $A_G/A_D = 10^{-4}$, $dI/dV = 1.85 \times 10^{-5} \pm 5.27 \times 10^{-6}$ S, $A_G/A_D = 10^{-3}$, $dI/dV = 2.12 \times 10^{-5} \pm 7.07 \times 10^{-6}$ S, $A_G/A_D = 10^{-2}$, $dI/dV = 1.26 \times 10^{-5} \pm 3.92 \times 10^{-6}$ S, $A_G/A_D = 10^{-1}$, $dI/dV = 1.56 \times 10^{-5} \pm 7.16 \times 10^{-6}$ S, $A_G/A_D = 10^{0}$, $dI/dV = 8.40 \times 10^{-5} \pm 1.97 \times 10^{-5}$ S, $A_G/A_D = 10^{1}$, $dI/dV = 9.10 \times 10^{-5} \pm 1.80 \times 10^{-5}$ S, $A_G/A_D = 10^{2}$, $dI/dV = 1.12 \times 10^{-4} \pm 5.22 \times 10^{-5}$ S.

C) Slope of saturation as a function of source/drain asymmetry and the gate area/drain contact area at the region of interest where contact area surpasses gate area for PEDOT:PSS-based channel. (W, L = 100 µm, A_G = 2500 µm^2, channel composition = PEDOT:PSS) V_G = 0.6 V and V_D = 0.6 V). A_D/A_S = 1, $dI/dV = 7.68 \times 10^{-4} \pm 1.72 \times 10^{-4}$ S, A_D/A_S = 1.05, $dI/dV = 8.29 \times 10^{-4} \pm 1.95 \times 10^{-4}$ S, A_D/A_S = 1.2, $dI/dV = 8.02 \times 10^{-4} \pm 2.00 \times 10^{-4}$ S, A_D/A_S = 5, $dI/dV = 7.65 \times 10^{-4} \pm 2.24 \times 10^{-4}$ S, A_D/A_S = 20, $dI/dV = 4.42 \times 10^{-4} \pm 1.19 \times 10^{-4}$ S, A_D/A_S = 80, $dI/dV = 2.11 \times 10^{-4} \pm 8.06 \times 10^{-5}$ S, A_D/A_S = 500, $dI/dV = 3.37 \times 10^{-5} \pm 7.64 \times 10^{-6}$ S.

D) Slope of saturation as a function of source/drain asymmetry and the gate area/drain contact area at the region of interest where contact area surpasses gate area for PEDOT:PEI/PSS-based channel (W, L = 100 µm, A_G = 2500 µm^2, channel composition = PEDOT:PEI/PSS) V_G = 0.6 V and V_D = 0.6 V). A_D/A_S = 1, $dI/dV = 1.19 \times 10^{-4} \pm 2.41 \times 10^{-5}$ S, A_D/A_S = 1.05, $dI/dV = 1.458 \times 10^{-4} \pm 4.12 \times 10^{-5}$ S, A_D/A_S = 1.2, $dI/dV = 1.50 \times 10^{-4} \pm 4.55 \times 10^{-5}$ S, A_D/A_S = 5, $dI/dV = 1.05 \times 10^{-4} \pm 2.81 \times 10^{-5}$ S, A_D/A_S = 20, $dI/dV = 3.61 \times 10^{-5} \pm 8.74 \times 10^{-6}$ S, A_D/A_S = 80, $dI/dV = 3.57 \times 10^{-5} \pm 2.56 \times 10^{-6}$ S, A_D/A_S = 500, $dI/dV = 2.47 \times 10^{-6} \pm 1.98 \times 10^{-6}$ S.

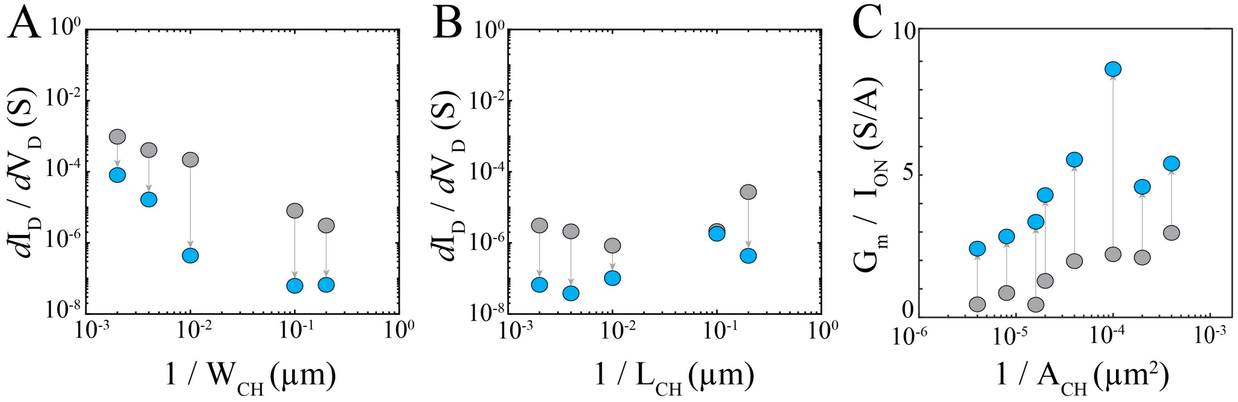

Supplementary Figure 6: Asymmetrical contact design principle is geometrically scalable.

A) Scatter plot showing the relationship between channel width and transistor saturation for a range of symmetric (grey) and asymmetric (1:100 asymmetry, blue) devices (L = 500 µm).

B) Scatter plot showing the relationship between channel width and transistor saturation for a range of symmetric (grey) and asymmetric (1:100 asymmetry, blue) devices (W = 5 µm).

C) Scatter plot demonstrating scalable improvement of normalized transconductance by ON current (I_{ON}) in saturation region. Blue and gray circles represent asymmetrical and symmetrical contact area devices, respectively.

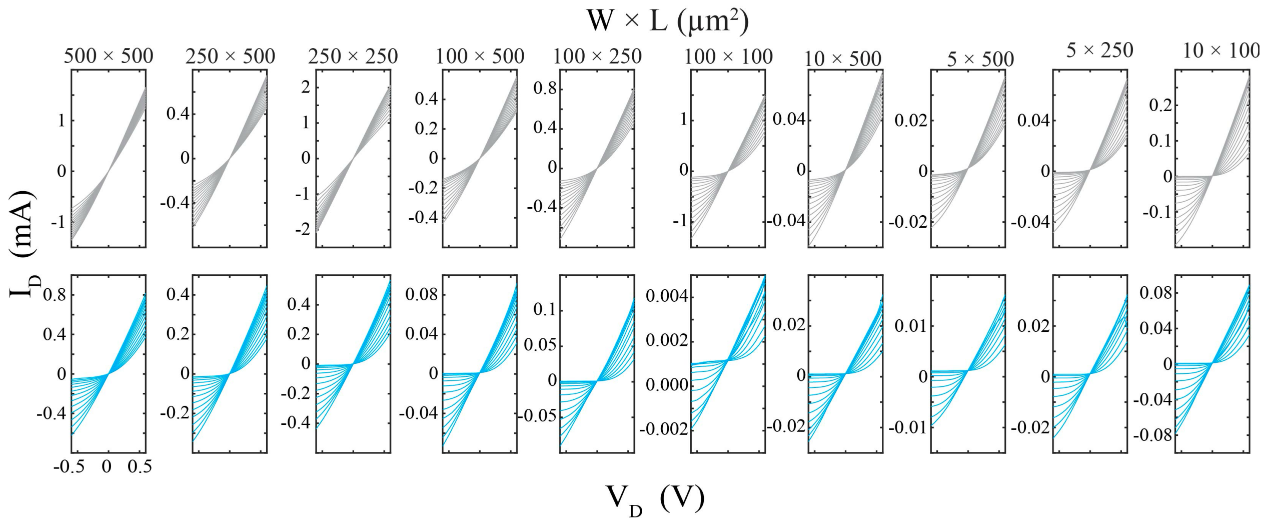

Supplementary Figure 7: Asymmetrical contact area design principle is scalable.

Improvement of current-voltage measurements for symmetric devices (top) compared to asymmetric contact area devices (bottom) for various channel geometries.

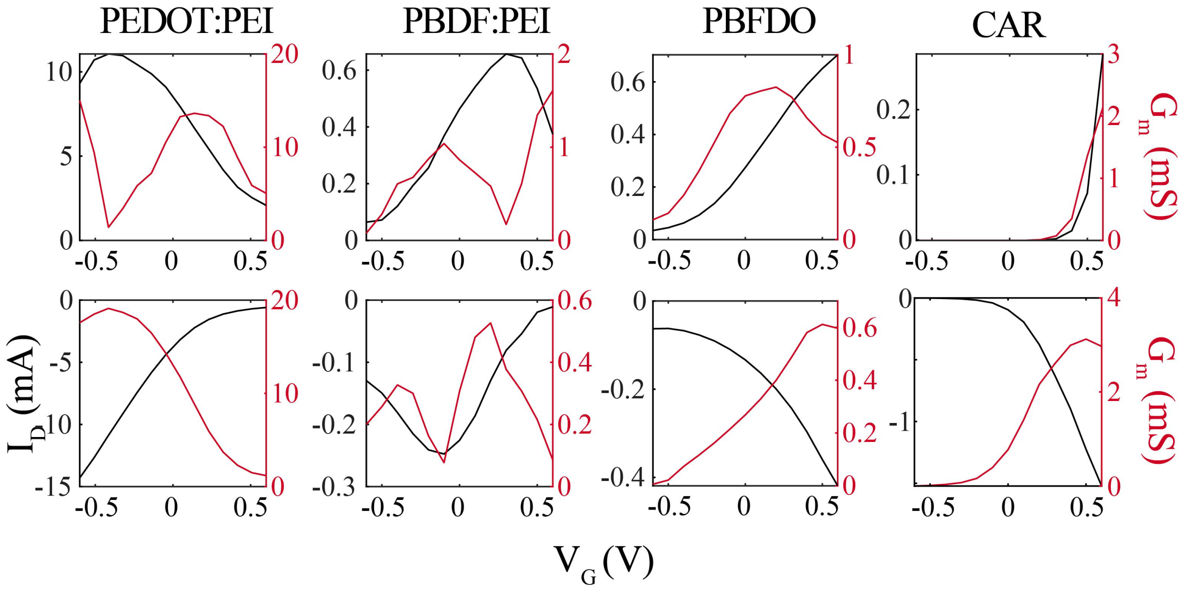

Supplementary Figure 8: Asymmetrical contact area design principle can create bipolar devices regardless of the electronic charge carrier used.

Transfer curves and transconductance of devices shown in **Figure 1G**.

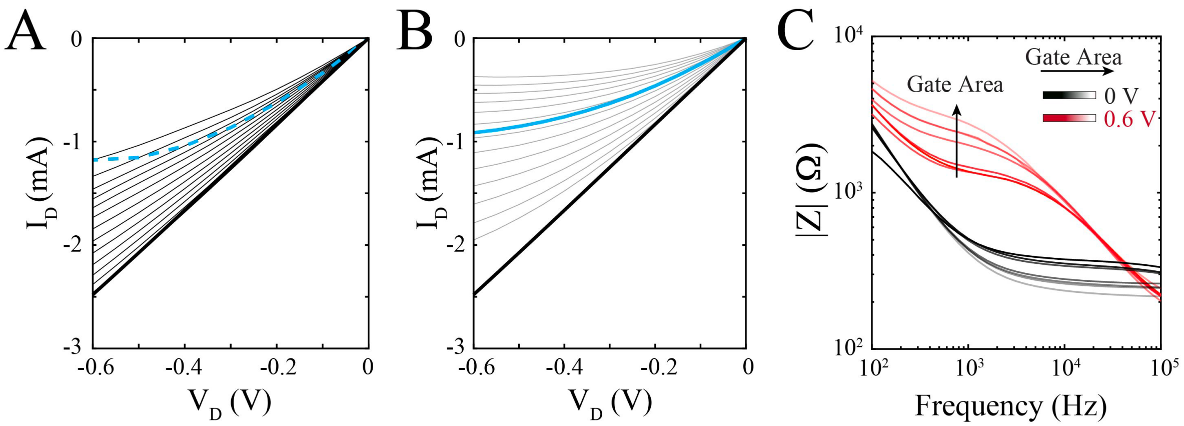

Supplementary Figure 9: Larger contact can effectively modulate the drain current without the presence of a gate electrode.

A) Output characteristics (gray curves) of a symmetric IGT (W, L = 100, 100 µm, V_D, V_G = –0.6 to 0.6 V). Black curve demonstrates I_D in the absence of a gate potential. Blue dashed curve represents each point on the IV curve where V_S-V_D = V_G (W, L = 100, 100 µm).

B) Output characteristics (gray curves) of an asymmetric IGT (W, L = 100, 100 µm, V_D, V_G = –0.6 to 0.6 V). Black curve demonstrates I_D of a symmetrical contact area-based IGT in the absence of gate electrode. Blue curve demonstrates I_D of an asymmetrical IGT in the absence of a gate.

C) Impedance spectroscopy of IGT channel under 0 V (black) and 0.6 V (red) gate biases. Color shade indicates the gate area, with lighter shades indicating larger gate areas.

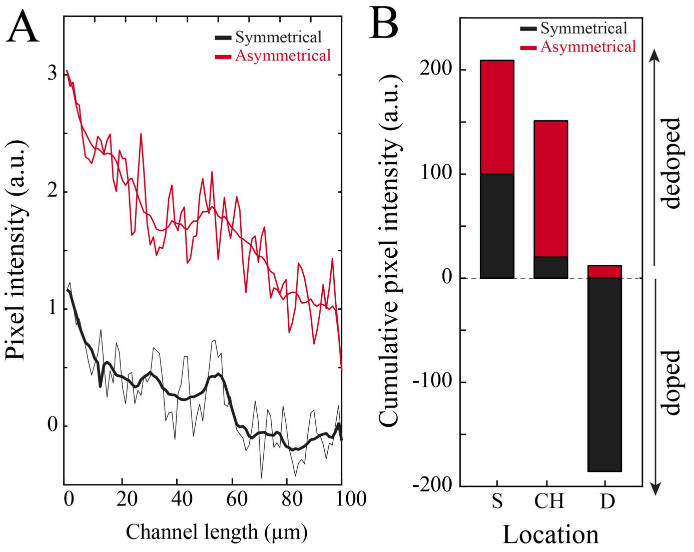

Supplementary Figure 10: Moving-front imaging reveals that asymmetrical contact design can mediate transistor channel dedoping.

A) Pixel intensity across the length of the channel in the symmetrical (black) and asymmetrical (red) cases. Thicker lines represent a moving average of the original data. Each data point represents 100 measured pixels in any given length position.

B) Cumulative pixel intensities representing the degree of dedoping at the source contact, channel area, and drain contact for a symmetrical (black) and asymmetrical (red) device.

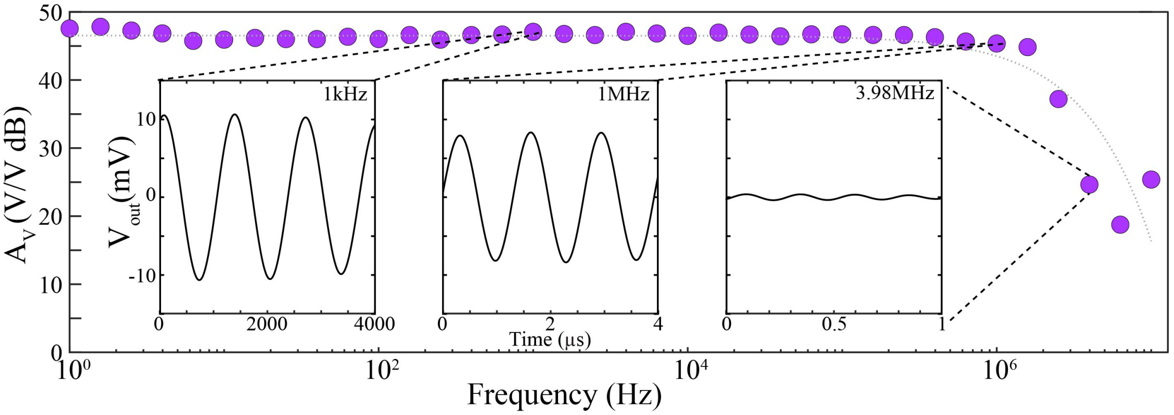

Supplementary Figure 11: Gain-bandwidth measurement procedure.

Sine wave outputs from three different frequencies in the gain bandwidth measurement showing a consistent gain (1kHz, 1MHz) below the frequency corner and a poor gain (3.98 MHz) above it. Input in all cases is a 100 µV peak-to-peak sine wave and output gain was calculated by finding the peak-to-peak voltage of the output signal and dividing output by input to obtain a V/V voltage gain measurement. Outputs were filtered with a Butterworth bandpass filter with frequency cutoffs at +-1% the frequency of interest and 0 dB passband gain.

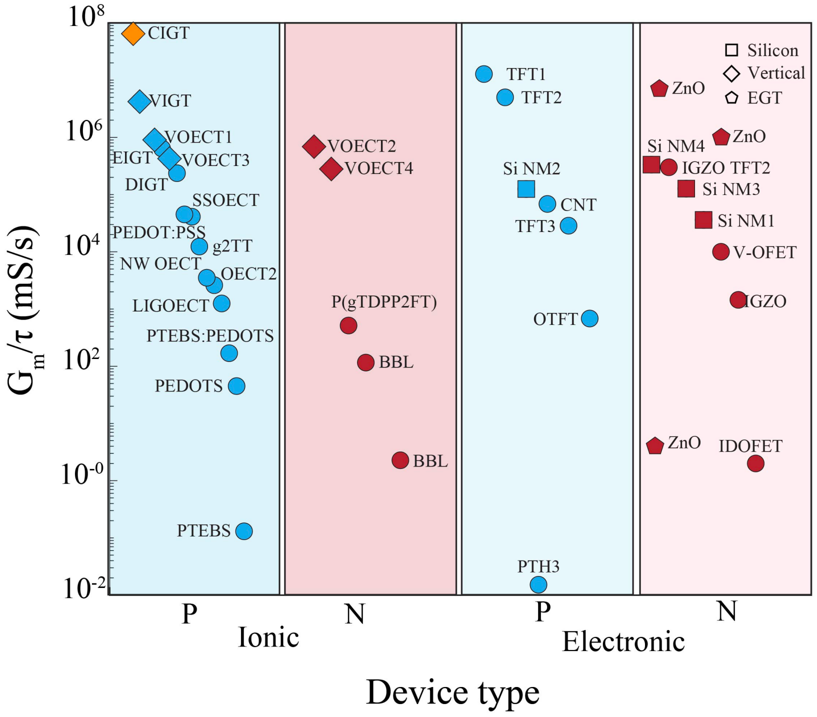

Supplementary Figure 12: Comparison of flexible transistor characteristics.

Ratio of transconductance and time constant of previously reported flexible transistors. Note: maximum transconductance, time constant, and W/L ratios were taken from the best performing reported device.

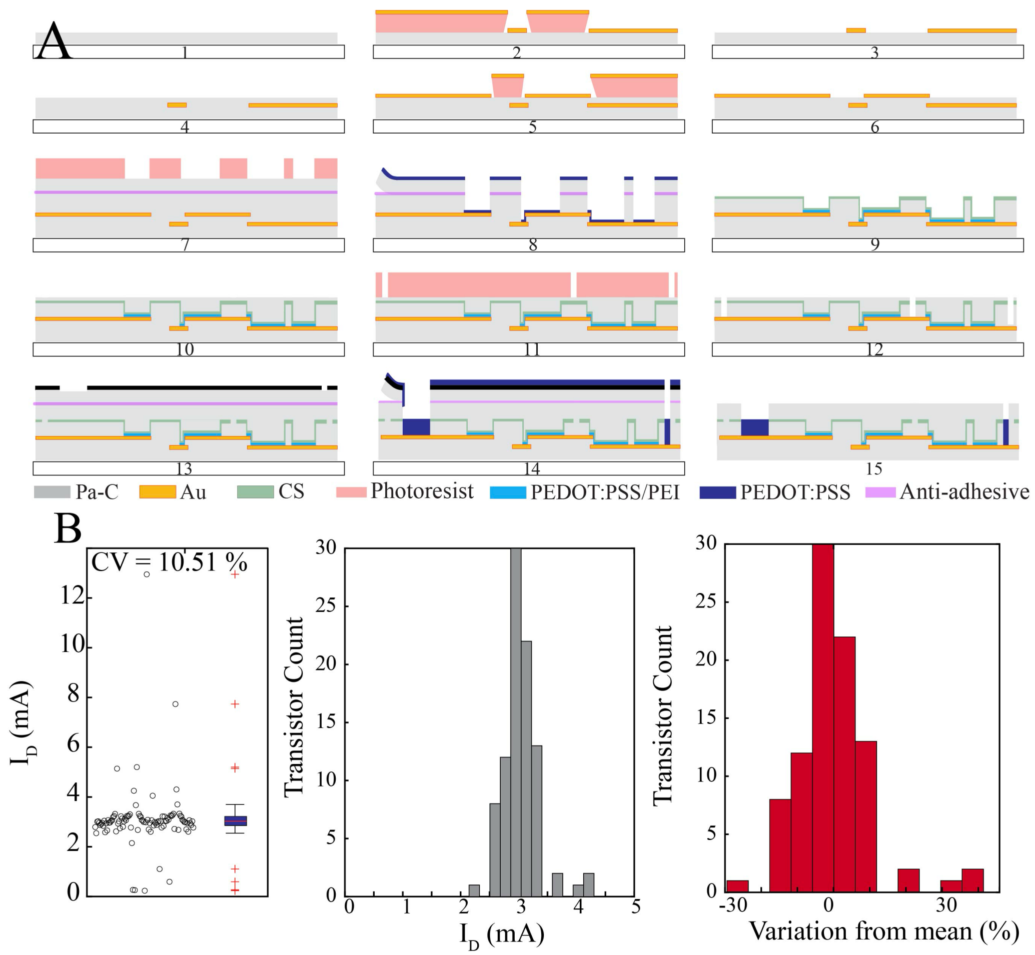

Supplementary Figure 13: Fabrication process and consistency of cIGT electrical properties.

A) Microfabrication process steps of vertical cIGTs. 1) CVD of Pa-C substrate on Si wafer. 2) Patterning and deposition of Ti/Au layer (M1) using e-beam thermal deposition. 3) Sonication assisted lift-off for patterning M1. 4) CVD of Pa-C with adhesion promoting silane on top of M1. 5) Patterning and deposition of Ti/Au layer (M2) using e-beam thermal deposition. 6) Sonication assisted lift-off for patterning M2. 7) CVD of Pa-C with adhesion promoting silane followed by anti-adhesion layer and additional CVD of Pa-C sacrificial layer with no silane and patterning of etching resist. 8) O_2 plasma etching to pattern channels, contacts, and gates; spin coating PEDOT:PSS solution and peeling off the sacrificial Pa-C for patterning of PEDOT:PSS. 9) Spin coating of PEI and chitosan. 10) CVD of Pa-C with adhesion promoting silane on top of chitosan. 11) Lithography to pattern chitosan layer. 12) O_2 plasma etching of chitosan followed by IPA rinse (only) to remove excess resist. 13) CVD of Pa-C with adhesion promoting silane followed by anti-adhesion layer, additional CVD of Pa-C sacrificial layer with no silane and Ti-based hard mask. 14) O_2 plasma etch followed by spin coating PEDOT:PSS and peel off patterning. 15) Water assisted removal from Si wafer.

B) Distribution of transistor ON current (left) with red markings denoting outliers and box plot marking interquartile ranges. A coefficient of variance (CV) of 10.51% and a yield of 91% were obtained from 100 measured devices. Histogram of devices classified by ON current showing a normal distribution (center), and by variation from mean (right).

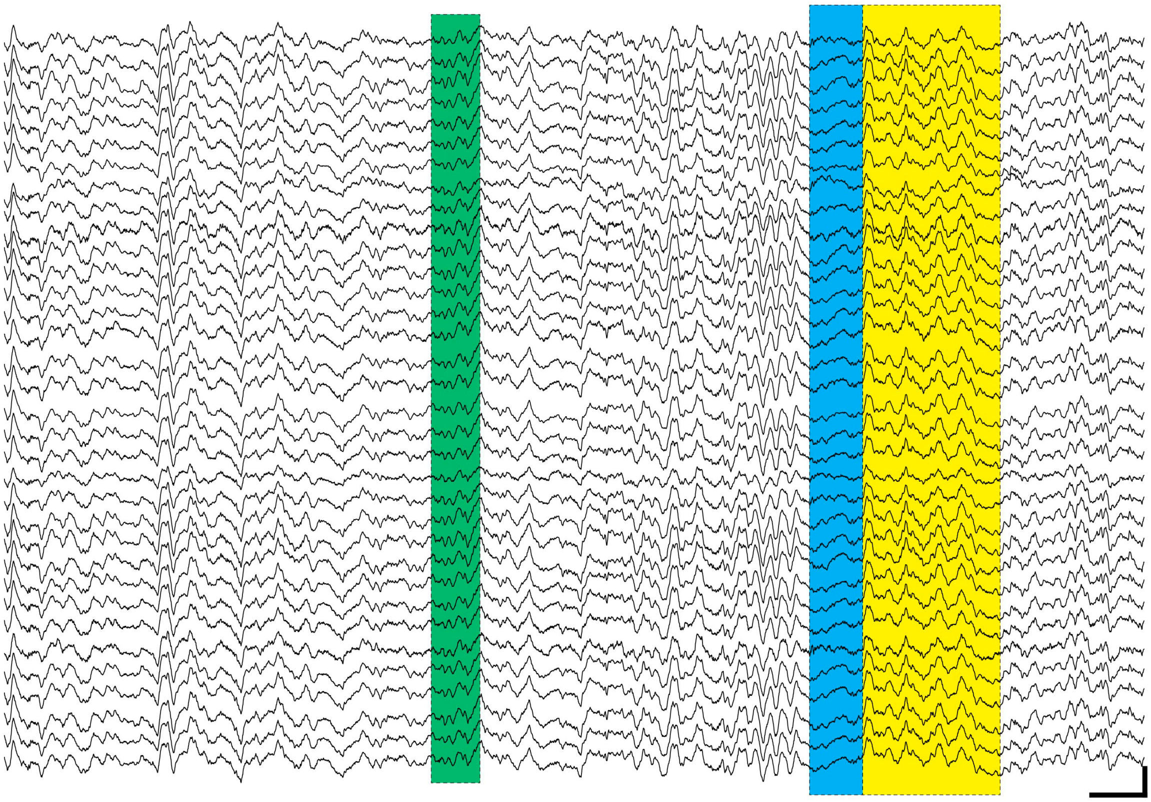

Supplementary Figure 14: Chronic recording of neural activity in freely moving rats. Multichannel LFP time-traces of posterior parietal cortex with visible gamma (green), delta (blue), and spindle (yellow) oscillations (scale bar, 200 ms, 250 μV).

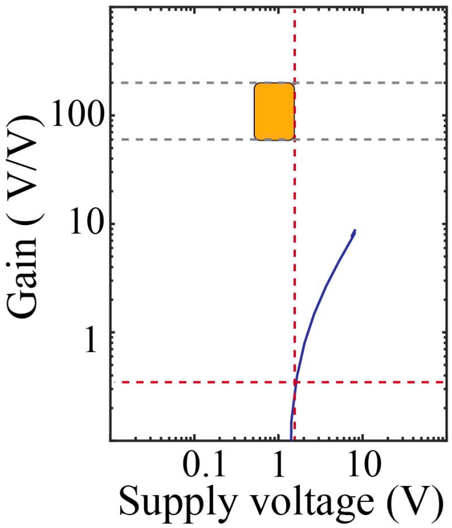

Supplementary Figure 15: IGT-based common source amplifier with a resistor load and symmetrical contacts requires high supply voltages to achieve acceptable voltage gain for fully implantable devices.

Estimated relationship between gain and supply voltage for a symmetrical IGT based on equations in **Supplementary Figure 3C**. Orange area represents the desired gain and supply voltages.

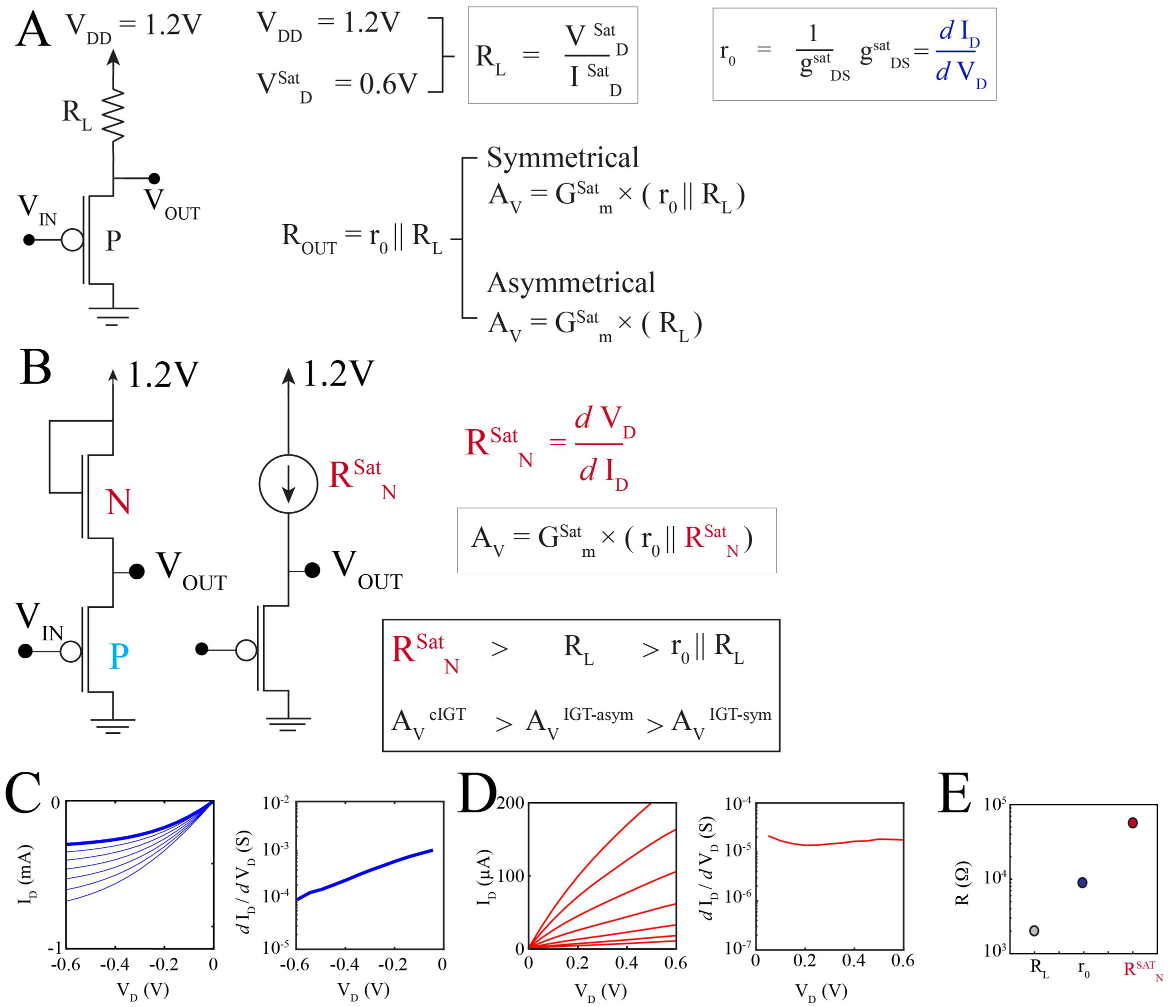

Supplementary Figure 16: Comparison of effective output resistance and gain between a resistor loaded and non-ideal constant current source-based common source amplifiers.
A) Schematic of a resistor load, common-source amplifier circuit (left) and the effect of saturation slope ($1/r_0$) on the overall gain.

B) Schematic of current source-based common-source amplifier circuit constructed using a 1st quadrant (N)-operating transistor with gate and drain terminals connected together (left). Relationship of voltage gain with the saturation slope ($1/R^{Sat}_N$) of the 1st quadrant-operating transistor. CIGT achieves the highest gain compared to a single transistor design.

C) Sample output characteristics (left) and saturation slope (right) of a 3rd quadrant-operating asymmetrical IGT device (W, L = 100 µm, $A_S/A_D = 100$).

D) Sample output characteristics (left) and saturation slope (right) of a 1st quadrant-operating asymmetrical IGT device (W, L = 100 µm, $A_D/A_S = 100$).

E) Resultant load resistance (R_L; $V_{DD} = 1.2V$), r_0 and resistance of the non-ideal constant current source constructed with a 1st quadrant-operating asymmetrical IGT device.

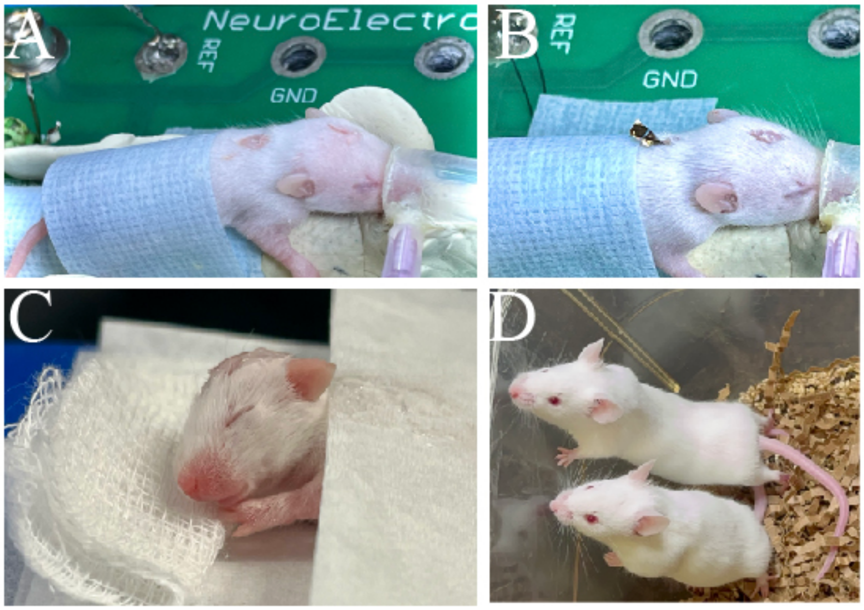

Supplementary Figure 17: Trocar-guided implantation of cIGT-based amplifiers in mouse pups.

A) Anesthetized mouse pup with incisions prepared at scalp and dorsal body areas.

B) Anesthetized mouse pup with implanted probe. Note contacts are still visible prior to insertion into subcutaneous space.

C) Post-surgical mouse pup recovering prior to a recording.

D) Implanted mouse as an adult (upper), indistinguishable from an unimplanted animal (lower).

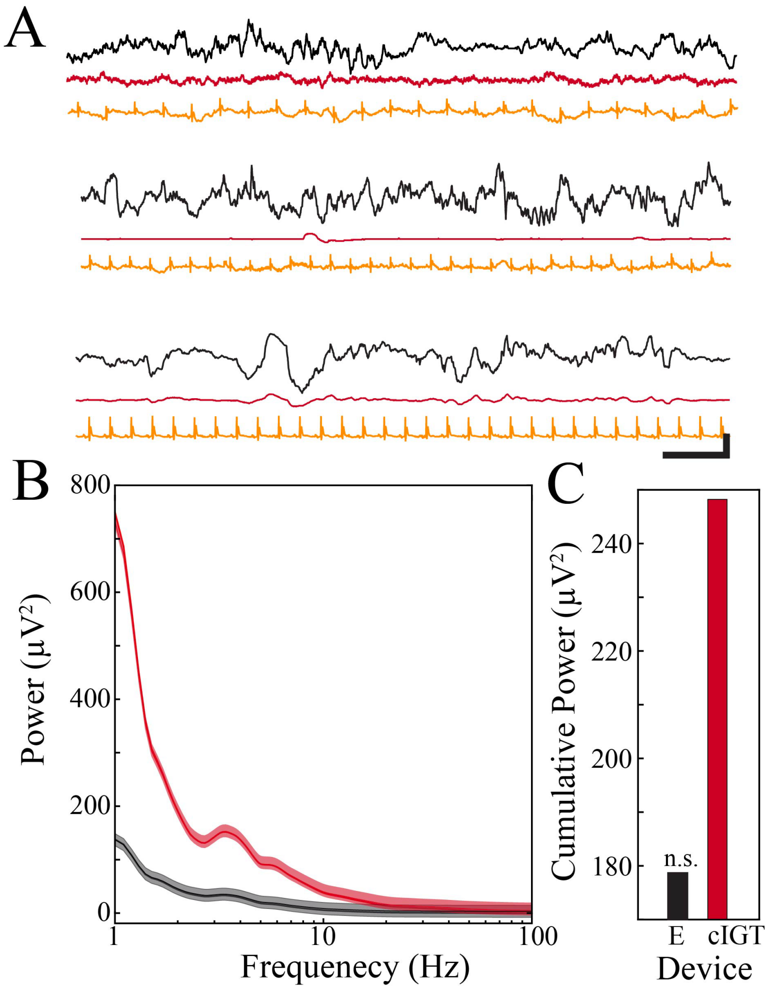

Supplementary Figure 18: Chronic acquisition of neural activity over the course of development.

A) Sample raw LFP traces comparing simultaneous recording acquired by cIGT-based amplifier (black) and conducting polymer electrode (red). Orange trace shows simultaneously acquired ECG and EMG activities to demonstrate lack of contamination of neural signals. These recordings were acquired from the same mouse on three different days: P9 (top), P10 (middle) and P12 (bottom) (scale bar, 1 s, 300 μV).

B) Comparison of power spectrum generated by an analytical wavelet transform of neural signals acquired by implanted cIGT (red) and conducting polymer-based electrode. Shaded areas represent mean ± standard error over 4000 data points.

C) Cumulative coupling power between low and high gamma signals acquired by cIGT (red) and conducting polymer-based electrode. Note that the conducting polymer-based electrode recording did not reveal significant low/high gamma coupling after Bonferroni-Holm correction.

P material	N material	τ^{max} (s)	G_m^{max} (mS)	Density (T/cm^2)	V_{DD} (V)
PEDOT:PSS	PEDOT:PSS	7.500E-08	4.85	2.581E+05	1
gDPP-g2T[45]	Homo-gDPP	4.250E-04	200	1.149E+02	0.7
bgDPP-g2T[73]	bHomo-gDPP	1.980E-04	99.1	2.222E+05	0.7
P(gTDPPT)[26]	P(gTDPP2FT)	1.750E-03	0.9	2.000E+02	0.8
P(g4 2T-T)[74]	BBL152	3.800E-04	0.044	2.000E+03	0.7
P(g2T-TT)[28]	P(C6NDI-T)	1.500E-04	1.5	1.250E+05	0.8
DPP-g2T[75]	IGZO	1.250E-01	2.5	2.500E+01	0.7
IGZO[76]	IGZO	1.100E-05	0.005	3.306E+04	6
TIPS-pentacene[77]	TIPS-pentacene	1.540E-03	0.0037	8.772E+00	100
CNT[78]	IGZO	1.100E-05	0.003	4.000E+04	5
CNT[79]	CNT	1.396E-07	0.006	2.000E+04	5
CNT[80]	a-IGZO	1.740E-06	0.05	5.000E+03	11
SI NM[81]	SI NM	1.500E-06	0.33	7.143E+03	4

Supplementary Table 1: Complementary transistor parameters from **Figure 2G.**

Device	τ (s)	G_m^{max} (mS)	G_m^{max}/τ (mS/s)	L (µm)	W (µm)	Type
CIGT	7.50E-08	4.85E+00	6.47E+07	5.00E-02	2.00E+00	P
VIGT[22]	9.20E-07	3.87E+00	4.21E+06	8.00E-01	2.00E+00	P
EIGT[20]	8.60E-06	5.49E+00	6.38E+05	3.00E+01	5.00E+00	P
DIGT[14]	2.80E-05	6.62E+00	2.36E+05	3.00E+01	1.20E+01	P
DOECT[13]	3.90E-05	1.60E+00	4.10E+04	1.00E+01	1.00E+01	P
g2TT[83]	6.40E-04	7.90E+00	1.23E+04	1.00E+01	1.00E+01	P
PTEBS:PEDOTS[84]	7.10E-02	1.20E+01	1.69E+02	2.00E+01	1.13E+03	P
PEDOTS[84]	3.60E-01	1.62E+01	4.50E+01	2.00E+01	1.13E+03	P
EGT1[85]	5.00E-05	2.00E-04	4.00E+00	1.00E+01	2.00E+02	N
BBL[86]	1.67E-01	3.80E-01	2.28E+00	3.00E+01	1.00E+03	N
PTEBS[84]	6.90E+00	9.00E-01	1.30E-01	2.00E+01	1.13E+03	P
PTH3[87]	1.00E-03	1.52E-05	1.52E-02	2.00E+01	2.00E+02	P
Si NM4[81]	1.50E-06	5.00E-01	3.33E+05	1.00E+01	3.30E+01	N
IGZO TFT[88]	6.92E-08	1.00E-04	1.45E+03	2.00E+01	1.00E+02	N
IGZO TFT2[89]	1.00E-05	3.00E+00	3.00E+05	2.00E+00	1.20E+03	N
V-OFET[90]	1.00E-07	1.00E-03	1.00E+04	3.00E-01	5.00E+00	N
Si NM1[91]	5.00E-06	1.80E-01	3.60E+04	4.00E+01	2.00E+02	N
Si NM3[92]	2.60E-06	3.30E-01	1.27E+05	1.38E+01	8.00E+01	N
EGT2[93]	5.00E-07	3.50E+00	7.00E+06	1.00E+01	2.00E+02	N
TFT1[94]	4.70E-08	6.00E-01	1.28E+07	6.00E-01	1.00E+02	P
TFT2[95]	4.00E-08	2.00E-01	5.00E+06	2.00E-01	5.00E+01	P
TFT3[96]	5.60E-07	1.60E-02	2.86E+04	1.20E-01	8.00E+01	P
EGT3[97]	1.00E-06	1.00E+00	1.00E+06	1.00E+01	2.00E+02	P
Si NM2[47]	4.00E-06	5.00E-01	1.25E+05	2.00E+01	2.00E+02	N
VOECT1[45]	4.25E-04	3.84E+02	9.04E+05	1.00E-01	3.00E+01	P
VOECT2[45]	3.66E-04	2.51E+02	6.86E+05	1.00E-01	3.00E+01	N
VOECT3[73]	1.98E-04	8.42E+01	4.25E+05	1.00E-01	1.00E+01	P
VOECT4[73]	1.54E-04	4.31E+01	2.80E+05	1.00E-01	1.00E+01	N
OECT1[74]	3.80E-04	4.40E-02	1.16E+02	1.00E+01	1.00E+02	N
OECT2[26]	4.60E-04	1.20E+00	2.61E+03	1.00E+01	1.00E+02	P
OECT3[26]	1.75E-03	9.00E-01	5.14E+02	1.00E+01	1.00E+02	N
CNT[79]	1.40E-07	9.60E-03	6.88E+04	2.00E+00	1.20E+01	P
SSOECT[98]	2.22E-04	1.00E+01	4.50E+04	2.00E+01	1.00E+02	P
LIGOECT[99]	1.00E-02	1.25E+01	1.25E+03	5.00E+02	4.00E+03	P
IDOFET[100]	1.00E-01	2.00E-01	2.00E+00	8.00E+01	1.94E+05	N
Ag NW OECT[101]	2.84E-04	1.00E+00	3.52E+03	2.00E+01	2.00E+03	P
OTFT[102]	4.40E-06	3.00E-03	6.82E+02	1.00E+01	1.60E+04	P

Supplementary Table 2: Flexible transistor parameters from **Supplementary Figure 12.**

Reference:

73. Kim, J. *et al.* Monolithically integrated high-density vertical organic electrochemical transistor arrays and complementary circuits. *Nature Electronics 2024 7:3* **7**, 234–243 (2024).
74. Wu, H. Y. *et al.* Influence of Molecular Weight on the Organic Electrochemical Transistor Performance of Ladder-Type Conjugated Polymers. *Advanced Materials* **34**, (2022).
75. Yao, Y. *et al.* Flexible complementary circuits operating at sub-0.5 V via hybrid organic-inorganic electrolyte-gated transistors. *Proc Natl Acad Sci U S A* **118**, e2111790118 (2021).
76. Naqi, M., Cho, Y. & Kim, S. High-Speed Current Switching of Inverted-Staggered Bottom-Gate a-IGZO-Based Thin-Film Transistors with Highly Stable Logic Circuit Operations. *ACS Appl Electron Mater* **5**, 3378–3383 (2023).
77. Lin, C. *et al.* Ambipolar Organic Field-Effect Transistors and Complementary Circuits Based on Single Crystals with Alcohol Treatment. *Adv Electron Mater* **8**, 2200557 (2022).
78. Chen, H., Cao, Y., Zhang, J. & Zhou, C. Large-scale complementary macroelectronics using hybrid integration of carbon nanotubes and IGZO thin-film transistors. *Nature Communications 2014 5:1* **5**, 1–12 (2014).
79. Zhong, D. *et al.* High-speed and large-scale intrinsically stretchable integrated circuits. *Nature 2024 627:8003* **627**, 313–320 (2024).
80. Zhang, J. *et al.* Ultra-Flexible Monolithic 3D Complementary Metal-Oxide-Semiconductor Electronics. *Adv Funct Mater* **33**, 2305379 (2023).
81. Song, E. *et al.* Flexible electronic/optoelectronic microsystems with scalable designs for chronic biointegration. *Proceedings of the National Academy of Sciences* **116**, 15398–15406 (2019).
82. Khodagholy, D. *et al.* High transconductance organic electrochemical transistors. *Nat Commun* **4**, 1–6 (2013).
83. Nielsen, C. B. *et al.* Molecular Design of Semiconducting Polymers for High-Performance Organic Electrochemical Transistors. *J Am Chem Soc* **138**, 10252–10259 (2016).
84. Zeglio, E. *et al.* Conjugated Polyelectrolyte Blends for Electrochromic and Electrochemical Transistor Devices. *Chemistry of Materials* **27**, 6385–6393 (2015).
85. Zare Bidoky, F. *et al.* Sub-3 V ZnO Electrolyte-Gated Transistors and Circuits with Screen-Printed and Photo-Crosslinked Ion Gel Gate Dielectrics: New Routes to Improved Performance. *Adv Funct Mater* **30**, 1902028 (2020).
86. Yang, C. Y. *et al.* A high-conductivity n-type polymeric ink for printed electronics. *Nature Communications 2021 12:1* **12**, 1–8 (2021).
87. Cho, J. H. *et al.* High-Capacitance Ion Gel Gate Dielectrics with Faster Polarization Response Times for Organic Thin Film Transistors. *Advanced Materials* **20**, 686–690 (2008).
88. Suresh, A. *et al.* Fast all-transparent integrated circuits based on indium gallium zinc oxide thin-film transistors. *IEEE Electron Device Letters* **31**, 317–319 (2010).
89. Rahaman, A., Chen, Y., Hasan, M. M. & Jang, J. A High Performance Operational Amplifier Using Coplanar Dual Gate a-IGZO TFTs. *IEEE Journal of the Electron Devices Society* **7**, 655–661 (2019).
90. Kleemann, H., Schwartz, G., Zott, S., Baumann, M. & Furno, M. Megahertz operation of vertical organic transistors for ultra-high resolution active-matrix display. *Flexible and Printed Electronics* **5**, 014009 (2020).
91. Viventi, J. *et al.* A conformal, bio-interfaced class of silicon electronics for mapping cardiac electrophysiology. *Sci Transl Med* **2**, (2010).
92. Fang, H. *et al.* Capacitively coupled arrays of multiplexed flexible silicon transistors for long-term cardiac electrophysiology. *Nature Biomedical Engineering 2017 1:3* **1**, 1–12 (2017).

93. Bidoky, F. Z. & Frisbie, C. D. Sub-3 V, MHz-Class Electrolyte-Gated Transistors and Inverters. *ACS Appl Mater Interfaces* **14**, 21295–21300 (2022).
94. Borchert, J. W. *et al.* Flexible low-voltage high-frequency organic thin-film transistors. *Sci Adv* **6**, (2020).
95. Zschieschang, U., Waizmann, U., Weis, J., Borchert, J. W. & Klauk, H. Nanoscale flexible organic thin-film transistors. *Sci Adv* **8**, 9845 (2022).
96. Haldar, T. *et al.* High-gain, low-voltage unipolar logic circuits based on nanoscale flexible organic thin-film transistors with small signal delays. *Sci Adv* **9**, (2023).
97. Cho, K. G. *et al.* Sub-Band Filling and Hole Transport in Polythiophene-Based Electrolyte-Gated Transistors: Effect of Side-Chain Length and Density. *Adv Funct Mater* **33**, 2303700 (2023).
98. Hou, K. *et al.* High Performance, Flexible, and Thermally Stable All-Solid-State Organic Electrochemical Transistor Based on Thermoplastic Polyurethane Ion Gel. *ACS Appl Electron Mater* **5**, 2215–2226 (2023).
99. Ren, G. *et al.* A laser-induced graphene-based flexible and all-carbon organic electrochemical transistor. *J Mater Chem C Mater* **11**, 4916–4928 (2023).
100. Nikolka, M. *et al.* Low-Voltage, Dual-Gate Organic Transistors with High Sensitivity and Stability toward Electrostatic Biosensing. *ACS Appl Mater Interfaces* **12**, 40581–40589 (2020).
101. Takemoto, A. *et al.* Fully Transparent, Ultrathin Flexible Organic Electrochemical Transistors with Additive Integration for Bioelectronic Applications. *Advanced Science* **10**, 2204746 (2023).
102. Kondo, M. *et al.* Highly-ordered Triptycene Modifier Layer Based on Blade Coating for Ultraflexible Organic Transistors. *Scientific Reports 2019 9:1* **9**, 1–9 (2019).
