# Supplementary material for: Spatial control of doping in conducting polymers enables complementary, conformable, implantable internal ion-gated organic electrochemical transistors
Source: Nat Commun. 2025 Jan 9;16:517. doi: 10.1038/s41467-024-55284-w (PMC11717955; doi:10.1038/s41467-024-55284-w)
Supplement: Supplementary file 3 — Solar Cell Reporting Summary [file 41467_2024_55284_MOESM3_ESM.pdf]

nature portfolio

Corresponding author(s): *Double-anonymous peer review submissions: write DAPR and your manuscript number here instead of author names.*

Solar Cells Reporting Summary

Nature Portfolio wishes to improve the reproducibility of the work that we publish. This form is intended for publication with all accepted papers reporting the characterization of photovoltaic devices and provides structure for consistency and transparency in reporting. Some list items might not apply to an individual manuscript, but all fields must be completed for clarity.

For further information on Nature Research policies, including our data availability policy, see Authors & Referees.

Experimental design

Please check the following details are reported in the manuscript, and provide a brief description or explanation where applicable.

1. Dimensions

Item	Yes/No	Description
Area of the tested solar cells	☐ Yes ☒ No	*Report the area of the tested solar cells (e.g. aperture area, active area).* NA
Method used to determine the device area	☐ Yes ☒ No	*Provide a description of the method and state where this information can be found in the text.* NA

2. Current-voltage characterization

Item	Yes/No	Description
Current density-voltage (J-V) plots in both forward and backward direction	☐ Yes ☒ No	NA
Voltage scan conditions	☐ Yes ☒ No	*Provide a description of the measurement conditions (e.g. scan direction, speed, dwell times).* NA
Test environment	☐ Yes ☒ No	*Provide a description of the test conditions (e.g. characterization temperature, atmosphere, humidity).* NA
Protocol for preconditioning of the device before its characterization	☐ Yes ☒ No	*Provide a description of the protocol.* NA
Stability of the J-V characteristic	☐ Yes ☒ No	*Provide a description of the method used. The stability of the J-V characteristic can be verified with time evolution of the maximum power point or with the photocurrent at maximum power point; see ref. 5 for details.* NA

3. Hysteresis or any other unusual behaviour

Item	Yes/No	Description
Description of the unusual behaviour observed during the characterization	☐ Yes ☒ No	*Provide a description of hysteresis or any other unusual behaviour observed during the characterization.* NA
Related experimental data	☐ Yes ☒ No	*Provide a description of the related experimental data.* NA

4. Efficiency

Item	Yes/No	Description
External quantum efficiency (EQE) or incident photons to current efficiency (IPCE)	☐ Yes ☒ No	*Provide a description of the technique used.* NA
A comparison between the integrated response under the standard reference spectrum and the response measure under the simulator	☐ Yes ☒ No	*State where this information can be found in the text.* NA

For tandem solar cells, the bias illumination and bias voltage used for each subcell

☐ Yes *Provide a description of the measurement conditions.*
☒ No NA

5. Calibration

Light source and reference cell or sensor used for the characterization

☐ Yes *Provide a description of the light source and reference cell or sensor.*
☒ No NA

Confirmation that the reference cell was calibrated and certified

☐ Yes *Identify the independent certification laboratory.*
☒ No NA

Calculation of spectral mismatch between the reference cell and the devices under test

☐ Yes *Provide a value of the spectral mismatch and/or a description of how it has been taken into account in the measurements.*
☒ No NA

6. Mask/aperture

Size of the mask/aperture used during testing

☐ Yes *Report the size of the mask/aperture.*
☒ No *Explain why this information is not reported/not relevant.*

Variation of the measured short-circuit current density with the mask/aperture area

☐ Yes *Report the difference in the short-circuit current density values measured with the mask and aperture area.*
☒ No NA

7. Performance certification

Identity of the independent certification laboratory that confirmed the photovoltaic performance

☐ Yes *Identify the independent certification laboratory.*
☒ No NA

A copy of any certificate(s)

☐ Yes *Certificate copies should be provided in the Supplementary information. Please state the supplementary item number.*
☒ No NA

8. Statistics

Number of solar cells tested

☐ Yes *Report how many solar cells have been tested, specifying the number of individual substrates.*
☒ No NA

Statistical analysis of the device performance

☐ Yes *State where this information can be found in the text.*
☒ No NA

9. Long-term stability analysis

Type of analysis, bias conditions and environmental conditions

☐ Yes *Provide a description of the type of analysis, bias conditions and environmental conditions (e.g. illumination type, temperature, atmosphere humidity, encapsulation method, preconditioning temperature, bias) for each long-term stability analysis carried out; see ref. 7 and 8 for details.*
☒ No NA
